# PRL3-DDX21 Transcriptional Control of Endolysosomal Genes Restricts Melanocyte Stem Cell Differentiation

**DOI:** 10.1016/j.devcel.2020.06.013

**Published:** 2020-08-10

**Authors:** Jeanette A. Johansson, Kerrie L. Marie, Yuting Lu, Alessandro Brombin, Cristina Santoriello, Zhiqiang Zeng, Judith Zich, Philippe Gautier, Alex von Kriegsheim, Hannah Brunsdon, Ann P. Wheeler, Marcel Dreger, Douglas R. Houston, Christopher M. Dooley, Andrew H. Sims, Elisabeth M. Busch-Nentwich, Leonard I. Zon, Robert S. Illingworth, E. Elizabeth Patton

**Affiliations:** 1 MRC Human Genetics Unit, MRC Institute of Genetics and Molecular Medicine, University of Edinburgh, Western General Hospital, Crewe Road South, Edinburgh EH4 2XU, UK; 2 Cancer Research UK Edinburgh Centre, MRC Institute of Genetics and Molecular Medicine, University of Edinburgh, Edinburgh EH4 2XU, UK; 3 Laboratory of Cancer Biology and Genetics, Center for Cancer Research, National Cancer Institute, National Institutes of Health, Bethesda, MD 20892, USA; 4 Stem Cell Program and Division of Hematology, Oncology, Boston Children’s Hospital and Dana Farber Cancer Institute, Howard Hughes Medical Institute, Harvard Medical School, Harvard Stem Cell Institute, Stem Cell and Regenerative Biology Department, Harvard University, Boston, USA; 5 Institute of Quantitative Biology, Biochemistry and Biotechnology, Waddington Building, King's Buildings, University of Edinburgh, Edinburgh EH9 3BF, UK; 6 Wellcome Sanger Institute, Hinxton CB10 1SA, UK; 7 Max-Planck-Institute for Developmental Biology, Department ECNV, Max-Planck-Ring 5, 72076 Tübingen, Germany; 8 Cambridge Institute of Therapeutic Immunology & Infectious Disease (CITIID), Jeffrey Cheah Biomedical Centre, University of Cambridge, Puddicombe Way, Cambridge CB2 0AW, UK; 9 Centre for Regenerative Medicine, Institute for Regeneration and Repair, The University of Edinburgh, Edinburgh BioQuarter, 5 Little France Drive, Edinburgh EH16 4UU, UK

**Keywords:** zebrafish, melanocyte stem cell, small-molecule screen, regeneration, PRL3, PTP4A3, transcription elongation, MITF, DDX21, melanoma

## Abstract

Melanocytes, replenished throughout life by melanocyte stem cells (MSCs), play a critical role in pigmentation and melanoma. Here, we reveal a function for the metastasis-associated phosphatase of regenerating liver 3 (PRL3) in MSC regeneration. We show that PRL3 binds to the RNA helicase DDX21, thereby restricting productive transcription by RNAPII at master transcription factor (MITF)-regulated endolysosomal vesicle genes. In zebrafish, this mechanism controls premature melanoblast expansion and differentiation from MSCs. In melanoma patients, restricted transcription of this endolysosomal vesicle pathway is a hallmark of *PRL3-high* melanomas. Our work presents the conceptual advance that PRL3-mediated control of transcriptional elongation is a differentiation checkpoint mechanism for activated MSCs and has clinical relevance for the activity of PRL3 in regenerating tissue and cancer.

## Highlights

• Prl3a prevents premature MSC differentiation in zebrafish regeneration
• PRL3 binds DDX21 to restrain transcription of MITF-regulated endolysosomal genes
• V-ATPase inhibitors rescue *prl3a*^*−/−*^ premature MSC differentiation in regeneration
• PRL3-regulated transcription controls MSC fate and characterizes *PRL3*-high melanomas

## Graphical Abstract

**Figure:**
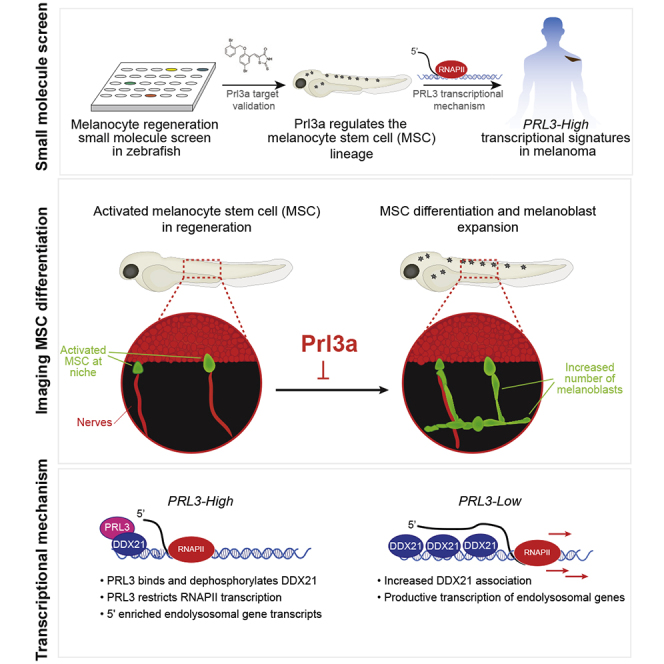

## Teaser

Activated melanocyte stem cells produce differentiated progenitors in regeneration. Johansson et al. reveal that the oncogene PRL3 regulates this process. PRL3 restricts productive transcription of MITF endolysosomal target genes bound by DDX21 in the melanocyte stem cell lineage to prevent premature melanoblast expansion during regeneration and in melanoma.

## Introduction

Stem cell and developmental pathways are often reactivated during regeneration and frequently mutated in cancers, providing a lens through which to resolve disease-associated biology across different forms of cancers (Sanchez-Vega et al., 2018). These pathways are conserved in zebrafish melanocyte development thereby enabling powerful chemical and genetic *in vivo* screens to discover new therapeutic candidates for melanoma (Cagan et al., 2019; van Rooijen et al., 2017).

Melanocytes are pigment cells derived from the neural crest or via a somatic stem cell population, and the melanocyte lineage gives rise to melanoma (Mort et al., 2015). In zebrafish, melanocytes emerge during early development from the *sox10*-expressing neural crest to form the embryonic stripe pattern (embryonic melanocytes), while a subpopulation of melanocyte stem cells (MSCs), also derived from the neural crest, establish a dormant niche at the site of the future dorsal root ganglia (Singh and Nüsslein-Volhard, 2015; Dooley et al., 2013a; Budi et al., 2011; Tryon et al., 2011; Hultman et al., 2009). MSCs are the source of a few late-stage embryonic melanocytes in the lateral stripe, adult stage melanocytes and regenerative melanocytes in embryos and adults (Iyengar et al., 2015; Yang and Johnson, 2006; Budi et al., 2011; Hultman and Johnson, 2010).

How zebrafish MSC activation is coupled with progenitor expansion and differentiation is not well understood. Erbb3 (EGFR [epidermal growth factor receptor] family) and Kit signaling pathways are required for MSC establishment at the dorsal root ganglia, and zebrafish embryos with *erbb3* mutations or treated with ERB inhibitors during early development are depleted for MSCs and are unable to regenerate sufficient melanocytes to pattern the embryonic or adult stripes (Dooley et al., 2013a; Budi et al., 2011; Johnson et al., 2011; Hultman et al., 2009). Following MSC establishment, the melanocyte master transcription factor (MITF; Mitfa in zebrafish) is essential for the proliferation and differentiation of MSC-derived melanocyte populations (Johnson et al., 2011). Additional MSC populations may be present in the zebrafish embryo, including a recently described ERB-dependent population associated with blood vessels and dependent on endothelin factors (Camargo-Sosa et al., 2019).

Here, we used both chemical and genetic melanocyte ablation approaches to trigger a MSC-mediated regenerative response and screened for small-molecule suppressors or enhancers of differentiation during regeneration. We discovered that the phosphatase PRL3 inhibits premature progenitor expansion and differentiation of the MSC lineage and that a PRL3 inhibitor leads to an increased rate of pigmented cell regeneration. *PRL3* is a member of the phosphatase of regenerating liver (*PRL*) gene family (Zeng et al., 1998), named because *PRL1* was first identified in regenerating liver (Mohn et al., 1991). *PRL3* mRNA is expressed in development and somatic tissues, but PRL3 protein translation is tightly regulated, and little PRL3 protein is present in somatic tissues (Thura et al., 2016; Lin et al., 2013; Maacha et al., 2013; Wang et al., 2010). PRL3 endogenous function remains largely unknown.

The PRLs are a unique class of protein tyrosine phosphatases (PTPs) with a broad spectrum of potential substrates (McParland et al., 2011; Al-Aidaroos and Zeng, 2010). PRL3 has a well-established role in cancer cell migration, is highly expressed in metastatic cancers, and is a marker of poor prognosis (Wei et al., 2018; Laurent et al., 2011; Al-Aidaroos and Zeng, 2010; Bardelli et al., 2003; Zeng et al., 2003; Saha et al., 2001). *PRL3* is a p53 target gene and cell-cycle regulator (Basak et al., 2008) and has various targets (Chong et al., 2019; Duciel et al., 2019; Zhang et al., 2017; Lin et al., 2013; Maacha et al., 2013; Basak et al., 2008). Supporting a role for the *PRL* family in regeneration, mutations in *PRL2* lead to a depletion of hematopoietic stem cells in mouse (Kobayashi et al., 2017, 2014). However, PRL3 has no previously known function in stem cell biology or regeneration. Here, we reveal an endogenous function for PRL3 in transcriptional elongation in both MSC regeneration and in melanoma.

## Results

### A Zebrafish Small-Molecule Screen Uncovers B4-Rhodanine as a Regulator of MSCs

**Figure 1:**
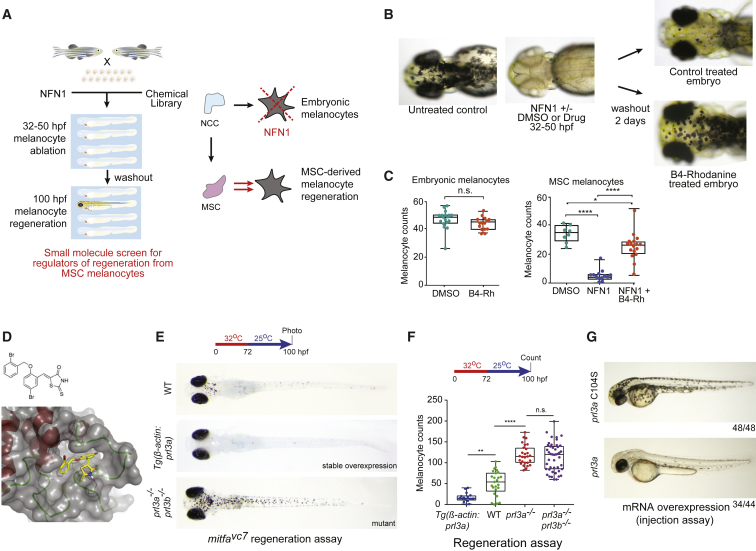
Prl3a Is an Inhibitor of Melanocyte Regeneration in Zebrafish (A) Schematic of a small-molecule screen for regulators of MSC-derived melanocytes in zebrafish. MSC, melanocyte stem cell; NCC, neural crest cells. (B) Images of zebrafish embryos treated with NFN1 ± DMSO or B4-Rh and after washout. (C) Quantification of zebrafish melanocytes during normal development (n.s., not significant, Student’s t test) or in a NFN1-regeneration assay (ANOVA using Tukey’s analysis; ^∗^p value = 0.0131; ^∗∗∗∗^p ≤ 0.0001). (D) Predicted binding of B4-Rh (yellow sticks) in the NMR model of PRL3 (gray transparent surface and secondary structure; red, helix; green, loop). Purple-dashed line: predicted hydrogen bond to E50. All other protein-ligand interactions are apolar. The ligand sits in a hydrophobic pocket formed by residues: V48, C49, W68, P69, A74, P75, P77, V80, A111, V113, and the methylene groups of the side chain of Q145. These residues, and E50, are conserved in zebrafish Prl3a. (E and F) (E) Images and (F) quantification of wild type, *Tg(β-actin:prl3a)*, *prl3a*^*−/−*^and *prl3* double mutant (*prl3a*^*−/−*^*; prl3b*^*−/−*^) zebrafish in a *mitfa*^*vc7*^ MSC regeneration assay (^∗∗^ < p < 0.01; ^∗∗∗∗^p < 0.0001; n.s., not significant; ANOVA using Tukey’s test). (G) RNA overexpression of *prl3a* and *prl3a* C104S in zebrafish embryos (50 hpf). See also Figures S1 and S2.

Whole genome sequencing has revealed that phosphatases are frequently mutated or lost in patients with melanoma (Hayward et al., 2017). Therefore, to identify potential regulators of activated MSCs, we treated zebrafish embryos with a library of small-molecule phosphatase inhibitors in the presence of NFN1. NFN1 is a 5-nitrofuran pro-drug that is activated by ALDH2 to selectively kill cells expressing *aldh2*, including zebrafish melanocytes (Sarvi et al., 2018; Zhou et al., 2012), although the cell death mechanism is not yet known in zebrafish. Embryonic melanocyte ablation triggers activation of a MSC regenerative program, enabling us to screen for regulators of the MSC (Figure 1A).

We treated zebrafish embryos with the Enzo Life Sciences SCREEN-WELL phosphatase library (33 phosphatase inhibitors) in the presence of our melano-cytotoxic compound NFN1 and followed melanocyte regeneration over time after washout (Figure 1A). None of the inhibitors prevented the melano-cytotoxic effects of NFN1 indicating that they did not interfere with NFN1 activity in embryonic melanocytes. As expected, embryos treated with NFN1 and DMSO (a solvent control) were depleted of embryonic melanocytes at 50 h post fertilization (hpf) and showed the initiation of melanocyte regeneration by 100 hpf (Figure 1B). By comparison, embryos treated with NFN1 and the compound B4-Rhodanine (B4-Rh) were also depleted of embryonic melanocytes at 50 hpf but showed dramatic melanocyte regeneration by 100 hpf (Figure 1B). Time-lapse imaging showed that melanocytes emerged *de novo* from within the tissue more quickly in B4-Rh treated embryos (Video S1).

Video S1. Regenerating Melanocyte Develop from Deep Precursors within the Embryo, Related to Figure 1

Next, we used a second melanocyte regeneration assay based on a genetic temperature sensitive mutation in *mitfa*, called *mitfa*^*vc7*^ (Zeng et al., 2015; Johnson et al., 2011; Taylor et al., 2011). These mutant zebrafish do not generate embryonic melanocytes at higher water temperatures because they lack Mitfa activity, and after 48–72 h embryonic melanocyte precursors are considered to have died. However, they are able to establish ERB-dependent MSCs and generate melanocytes from the MSC lineage when Mitfa activity is restored at lower water temperatures (Johnson et al., 2011). We found that B4-Rh treatment during MSC establishment lead to an increase in melanocyte regeneration when Mitfa activity was restored at lower water temperatures in *mitfa*^*vc7*^ zebrafish (Figures S1A and S1B). Thus, B4-Rh effectively enhanced melanocyte regeneration in both chemical and genetic regeneration assays suggesting B4-Rh is a *bona fide* regulator of the MSC lineage.

B4-Rh induced the strongest regeneration phenotype in our screen with no effect on direct-developing melanocytes (Figure 1C). Adult melanocyte regeneration is also dependent on MSCs established during development (Mort et al., 2015). We found B4-Rh increased melanocyte regeneration from an unpigmented precursor in an adult tail fin regeneration assay (Rawls and Johnson, 2000) (Figures S1C–S1E). This indicates that B4-Rh can regulate melanocyte regeneration from the MSC in both embryonic and adult zebrafish.

B4-Rh is a highly reactive compound, which potently inhibits PRL3 (Ahn et al., 2006). Molecular modeling and automated docking predicted that B4-Rh binds and inhibits the PRL3 phosphatase site in a hydrophobic pocket that is conserved in zebrafish (Figure 1D). Given the specificity of B4-Rh for the MSC lineage and the novelty of B4-Rh and its target PRL3 in the stem cell response, we chose to focus on B4-Rh and PRL3 as novel regulators of the MSC lineage.

### Prl3a Inhibits Melanocyte Regeneration from the MSC Lineage

We hypothesized that Prl3 is targeted by B4-Rh in zebrafish and is required in the activated MSC lineage during regeneration. To test this, we generated *prl3* genetic loss-of-function and overexpression mutants and assayed their phenotypes in regeneration. Zebrafish have two *prl3* genes (*prl3a* and *prl3b*); Prl3a is the most similar to human at the protein level (87% versus 74% protein identity) (Figure S2A). Similar to the effects of B4-Rh, a TALEN *prl3a* mutant that we generated (as well as morpholino knockdown) had no overt embryonic melanocyte phenotype but had increased melanocyte regeneration in the *mitfa*^*vc7*^ background (Figures 1E, 1F, and S2B–S2E). B4-Rh treatment of the *prl3a* mutant did not further increase this phenotype, supporting the concept that B4-Rh is acting through Prl3a (Figure S2F). In contrast, a *prl3b* CRISPR mutant had no detectable embryonic or MSC melanocyte phenotype either on its own, or in addition to the *prl3a* mutant (Figures 1E, 1F, and S2D; data not shown) suggesting that Prl3b does not have unique functions in MSC differentiation. Thus, we focused our efforts on investigating *prl3a* in the MSC response.

Next, we overexpressed *prl3a* mRNA in zebrafish embryos and found that this inhibited melanocyte development (Figure 1G). This phenotype was lost upon mutation of the catalytic cysteine to serine (C104S) (Figure 1G), indicating Prl3a phosphatase activity is required to inhibit melanocyte development. To overexpress *prl3a* throughout the embryo, we generated homozygous *Tg(β-actin:prl3a)* transgenic zebrafish in the *mitfa*^*vc7*^ background (Figure S2G). Ubiquitous overexpression of *prl3a* partially inhibited development of embryonic melanoblasts and, importantly, suppressed melanocyte regeneration from the stem cell lineage (Figures 1E, 1F, S2H, and S2I).

### Loss of Prl3 Activity Prematurely Increases MSC-Derived Melanoblast Populations

**Figure 2:**
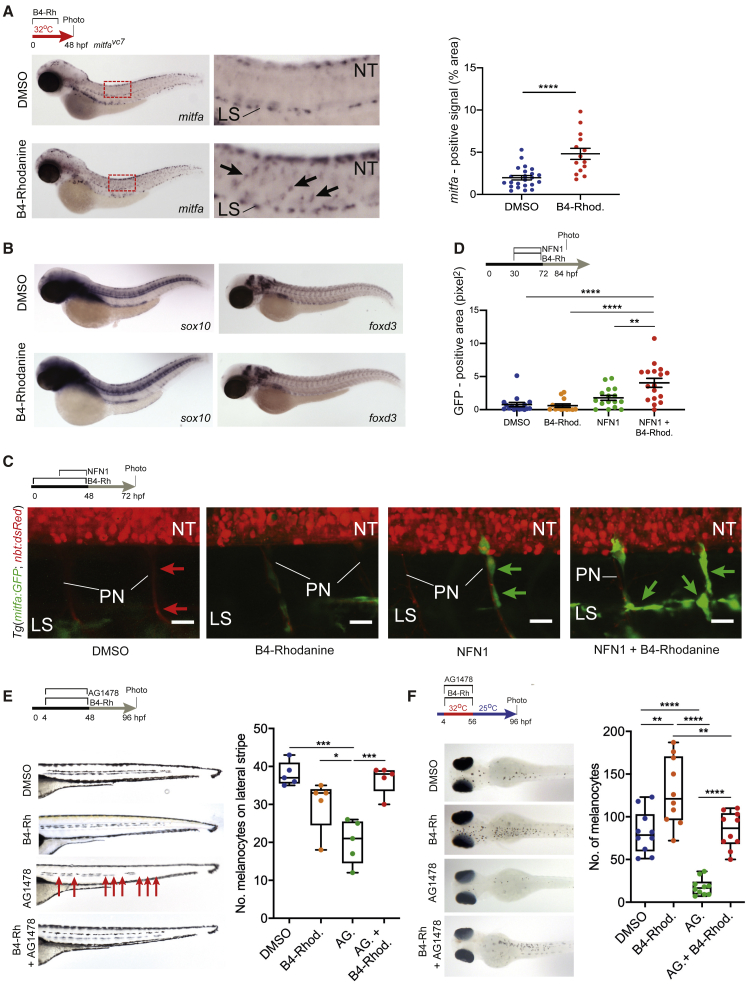
Inhibition of Prl3 Leads to Premature Melanoblast Expansion during Regeneration (A) *mitfa in situ* hybridization of DMSO and B4-Rh-treated *mitfa*^*vc7*^ zebrafish. Red box: zoomed region. Arrows: *mitfa+* melanoblasts. LS, lateral stripe; NT, neural tube. Percentage area covered by *mitfa*-positive staining per embryo quantified (^∗∗∗∗^p < 0.0001, t = 4.670, df = 35, unpaired, two-tailed t test). Line and error bars, mean ± SEM (DMSO, n = 23; B4-Rh, n = 14 embryos). (B) *sox10* (DMSO, n = 21; B4-Rh, n = 16) and *foxd3* (DMSO, n = 10; B4-Rh, n = 10) RNA *in situ* hybridization of treated *mitfa*^*vc7*^ zebrafish. (C) Confocal imaging of the MSC lineage during treatments specified. *Tg(mitfa:GFP)* marks regenerating melanoblasts (green arrows). *Tg(nbt:dsRED)* marks neural tube and axons (red arrows). n = 3 zebrafish per condition. NT, neural tube; PN, peripheral nerves; LS, lateral stripe. Maximum projection. Stack height: 30 μm. Scale bar: 20 μm. (D) Quantification of area of GFP expression (pixels^2^) on individual peripheral nerves of 84 hpf *Tg(mitfa:GFP, nbt:dsRED)* zebrafish embryos, 12-h post-washout treatment specified. B4-Rhod, DMSO, NFN1, and combination, n = 3 embryos; B4-Rhod control, n = 2 embryos. Line and error bars, mean ± SEM ^∗∗^p = 0.0039, ^∗∗∗∗^p = < 0.0001, ANOVA using Tukey’s analysis. (E) Lateral stripe melanocytes following specified treatments. AG.: AG1478. B4-Rhod: B4-Rhodanine. Red arrows: missing melanocytes; faint melanocytes from opposite side of the body are also visible. Quantification of melanocytes on the lateral stripe of embryos at 96 hpf, ^∗^p = 0.0364; ^∗∗∗^p = 0.0003 and 0.0006 for DMSO versus AG. and AG. versus AG. + B4-Rhod, respectively, ANOVA using Tukey’s analysis. (F) Melanocyte regeneration in *mitfa*^*vc7*^ mutant embryos following specified treatments. AG.: AG1478. B4-Rhod or B4-Rh: B4-Rhodanine. Quantification of dorsal stripe melanocytes per embryo 40-h post-washout and *mitfa* activation, ^∗∗^p = 0.0013, 0.0023 for DMSO versus B4-Rhod and B4-Rhod versus AG + B4-Rhod, respectively; ^∗∗∗∗^p < 0.0001, ANOVA using Tukey’s analysis.

To determine the developmental stage altered by B4-Rh, we treated *mitfa*^*vc7*^ zebrafish embryos (primed to regenerate) with B4-Rh and found that it led to an increase in *mitfa*-expressing cells (Figure 2A), but not of *sox10* or *foxd3*-expressing cells (Figure 2B). These data indicate that B4-Rh-mediated inhibition of Prl3a activity impacts upon melanocyte progenitor (melanoblast) development from MSCs during regeneration and not earlier in the neural crest lineage.

Next, we used live-imaging to examine *Tg(mitfa:GFP;nbt:dsRED)* zebrafish embryos during melanocyte regeneration (Dooley et al., 2013a) (Figures 2C and 2D). *mitfa:GFP* enables visualization of melanoblasts, whereas *nbt:dsRED* enables visualization of the neural tube and peripheral nerves, helping establish the location of the MSC niche (Dooley et al., 2013a). DMSO or B4-Rh treatment had no effect on *mitfa:GFP* expression in non-regenerating zebrafish. However, NFN1 treatment led to an increase in GFP+ cells at the MSC niche, indicating a regeneration response. Strikingly, embryos treated with NFN1 and B4-Rh showed a strong increase in GFP+ cells in the MSC niche and along motor axons (Figures 2C and 2D). These data show that inhibiting Prl3a causes premature expansion of melanoblasts during regeneration from the MSCs and reveal an activity for Prl3 in a stem cell lineage.

We wanted to understand if Prl3 functions before or after ERB signaling in the MSC lineage. Treatment of wild-type embryos with the ERB inhibitor AG1478 leads to gaps in the embryonic melanocyte lateral stripe (Hultman and Johnson, 2010). This assay enables us to test B4-Rh activity on the MSCs in a non-regenerating context. We found that the PRL3 inhibitor rescued the loss of the melanocytes in the lateral stripe of AG1478 treated embryos (Figure 2E). Next, we tested the PRL3 inhibitor in the *mitfa*^*vc7*^ regeneration assay with AG1478 (Figure 2F). ERB inhibitor treatment prevents melanocyte regeneration in the *mitfa*^*vc7*^ regeneration assay, indicating that regeneration in the *mitfa*^*vc7*^ mutant depends on ERB-dependent MSCs (Johnson et al., 2011). Co-treatment of B4-Rh and AG1478 rescued the number of regenerating melanocytes in the *mitfa*^*vc7*^ regeneration assay (Figure 2F). Together, these two independent MSC assays provide evidence that PRL3 inhibition rescues the dependency on ERB signaling, suggesting that PRL3 activity acts downstream of the ERB pathway in the MSC lineage.

### Prl3a Interacts with the RNA Helicase Ddx21

**Figure 3:**
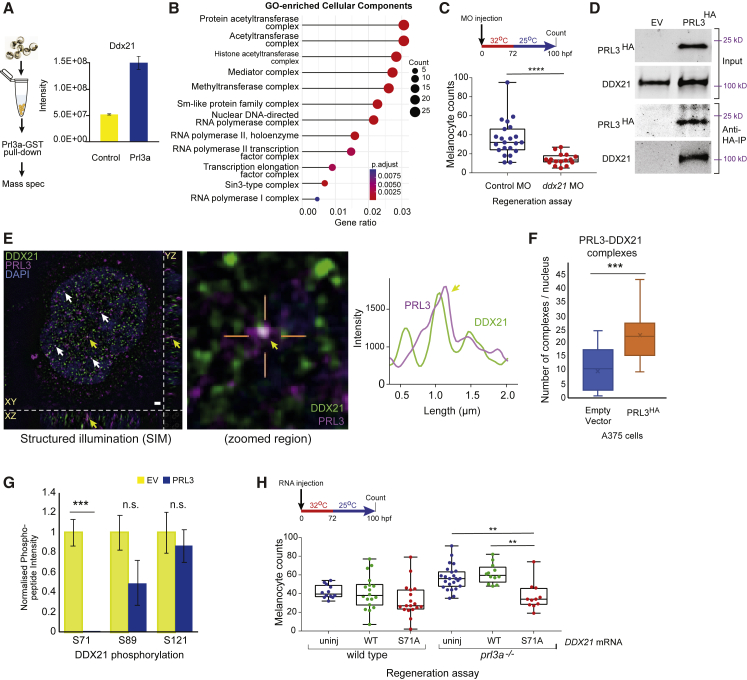
Prl3a Interacts with Ddx21 in Zebrafish and Melanoma Cell Nuclei (A) Experimental overview and intensity quantification of Ddx21 peptides from Prl3a-GST pull-downs. Bars and error bars are mean ± SEM. See also Table S1. (B) Cellular component GO-enrichment analysis of Prl3a-interacting proteins identified by co-immunoprecipitation and mass-spectrometry (adjusted p values: Benjamini-Hochberg test). (C) Quantification of melanocytes in a *mitfa*^*vc7*^ regeneration assay following control or *ddx21* morpholino injection, ^∗∗∗∗^p < 0.0001 Student's t test. (D) Co-immunoprecipitation of human HA-tagged PRL3 protein from A375 cells: empty vector (EV) and PRL3-expressing stable transfected cells. (E) Structured illumination microscopy (SIM) of endogenous PRL3 (magenta) and DDX21 (green) in C092 melanoma cells. Scale bar, 1 μm. Co-localization indicated (arrows); zoomed image shows PRL3-DDX21 co-localization with line scan, and intensity plot profile of line scan showing signal overlap (yellow arrow). DAPI: blue. See also Figure S3. (F) Quantification of PRL3-DDX21 complexes per nucleus identified by SIM in A375 melanoma cells expressing empty vector or HA-tagged PRL3 (^∗∗∗^p < 0.001, error bars: x indicates the mean; Student's t test). See also Figure S3. (G) Mass spectrometry of DDX21 phosphorylation sites in EV control cells (n = 5 samples) and cells expressing PRL3 (n = 7 samples) (^∗∗∗^p < 0.001; n.s., not significant; Bars and error bars are mean ± SEM; Student's t test). See also Table S2. (H) Quantification of regenerating melanocytes in wild-type or *prl3a* mutant *mitfa*^*vc7*^ embryos injected with mRNA encoding DDX21 (WT) or DDX21 S71A (S71A), or an uninjected control (uninj) (^∗∗^p < 0.01, ANOVA using Tukey’s analysis).

To explore Prl3a targets during zebrafish melanoblast development we generated a Prl3a-GST fusion protein, isolated interacting partners from zebrafish cell extract, and performed mass spectrometry. One of the strongest interacting partners for Prl3a identified was the DEAD box protein and RNA helicase Ddx21 (Figure 3A; Table S1). DDX21 has emerged as a central regulator of Pol I-dependent ribosome biogenesis and of Pol II transcriptional elongation and a sensor of nucleotide stress (Calo et al., 2018, 2015; Santoriello et al., 2020). Notably, we found that Prl3a bound many proteins involved in transcription control, including components of the acetyltransferase complexes, the mediator complex, RNA polymerase I and II, and the transcription elongation factor complex (Figure 3B).

To address if the Prl3a-Ddx21 interaction was relevant to the MSC regeneration response, we injected zebrafish embryos with a *ddx21* morpholino. As reported by others (Calo et al., 2018), we found no effect on embryonic melanocytes after *ddx21* knockdown. In contrast, we found *ddx21* to be required for the MSC regeneration response (Figure 3C). Given the strong interaction between Prl3a and Ddx21 in zebrafish extracts coupled with the requirement for Ddx21 in melanocyte regeneration, we chose to pursue Ddx21 as a novel Prl3a interacting partner.

To address if the zebrafish Prl3a-Ddx21 interaction is conserved in human cells, we generated A375 melanoma cells that stably express human PRL3 fused to HA. A375 cells express low levels of endogenous *PRL3* (Figures S3A and S3B), and *PRL3* overexpression had no discernible effect on growth or cell-cycle stages (Figures S3C–S2E). We found that DDX21 co-immunoprecipitated with PRL3-HA (Figure 3D). To study the subcellular localization of the PRL3-DDX21 interaction, we performed structured illumination microscopy (SIM), a super-resolution microscopy technique that enables resolution beyond conventional methods (Figure S3F). In cells that express high endogenous PRL3 (C092 melanoma cells), we found that PRL3 and DDX21 co-localized as punctate clusters in the nucleoplasm (Figures 3E, S3F, and S3G). Further, we found these foci were greatly enriched in the nuclei of A375 melanoma overexpressing PRL3-HA (Figure 3F).

DDX21 is predicted to be a highly phosphorylated protein, and phosphorylation may regulate DDX21 localization between the nucleus, nucleolus, and cytoplasm (Calo et al., 2018; Mialon et al., 2008). Using mass spectrometry, we found phosphorylation of DDX21 at sites S71, S89, and S121 (Figure 3G). S71 phosphorylation was strongly reduced in A375 cells expressing PRL3-HA, suggesting that PRL3 activity regulates DDX21 phosphorylation (Figure 3G; Table S2). To test if these sites had activity *in vivo*, we injected zebrafish embryos with human *DDX21* and *DDX21(S71A)* mRNA in the *mitfa*^*vc7*^ regeneration assay and showed *DDX21(S71A)* selectively reduced regeneration in the *prl3a* mutant but not *prl3a* wild-type embryos (Figure 3H). These data indicate that DDX21(S71A) functions in a dominant manner to rescue the *prl3a* mutant. Together, these data suggest that DDX21 de-phosphorylation is the primary function for Prl3a activity in melanocyte regeneration and that S71 is a critical target residue on DDX21 for PRL3.

### PRL3 Impairs Transcriptional Elongation of Endolysosomal Biogenesis Genes

**Figure 4:**
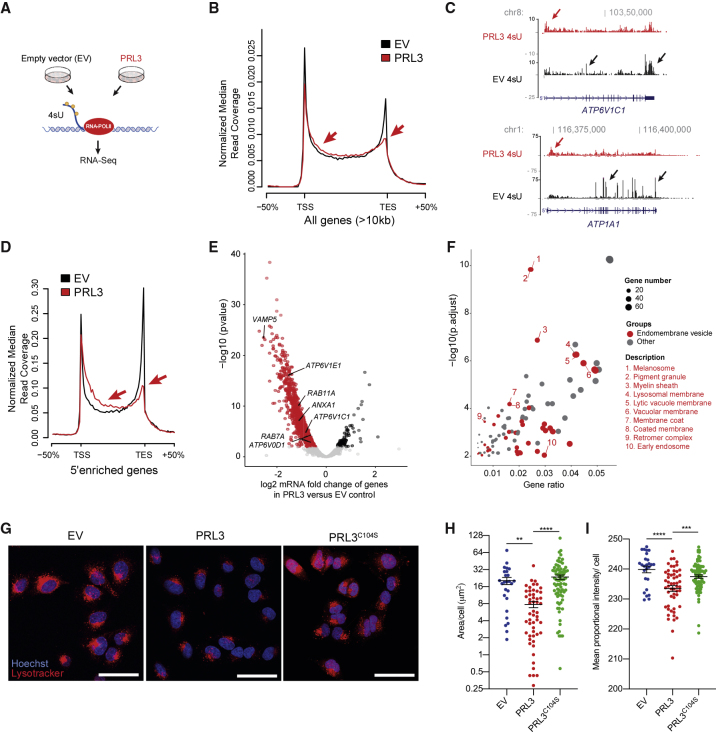
PRL3 Restrains Transcriptional Elongation of Endomembrane Vesicle Genes (A) Schematic of 4sU protocol to detect nascent RNA transcripts. (B) 4sU transcriptional profile for long genes in EV (empty vector) versus *PRL3* over-expressing cells (PRL3). TSS, transcription start site; TES, transcription end site. Arrows indicate accumulation of transcripts to the 5′ end and loss of transcripts in the 3′ end of *PRL3-*expressing cells (C) 4sU RNA-seq transcript coverage for *ATP6V1C1* and *ATP1A1* nascent transcripts. In control EV cells (black) most transcripts align with exons (black arrows), whereas transcripts in *PRL3* expressing cells (red) accumulate are enriched at the 5′ end of the gene (red arrows). (D) 4sU transcriptional profile for 5′-enriched genes in control and *PRL3*-expressing cells. Accumulation of transcripts to the 5′ end and a decrease at the 3′ end of the gene body (red arrows) in PRL3-expressing versus control EV cells. (E) Volcano plot of differentially expressed genes in PRL3 versus EV control A375 cells by DESeq2-analysis using 4sU nascent RNA-seq data. See also Table S3. (F) Bubble plot of GO cellular components in the *PRL3*-induced 5′ enriched genes. Endomembrane vesicle components highlighted in red. (G) A375 melanoma cells expressing EV, PRL3, and PRL3 (C104S) stained with LysoTracker (red) and hoechst (blue). Scale bars: 50 μm. (H) Quantification of the area of LysoTracker-positive vesicles per cell. ANOVA using Tukey’s analysis, ^∗∗^p = 0.0015; ^∗∗∗∗^p < 0.0001. Line and error bars, mean ± SEM. (I) Quantification of the proportional mean intensity of LysoTracker-positive vesicles per cell. ANOVA using Tukey’s analysis, ^∗∗∗^p = 0.0005; ^∗∗∗∗^p < 0.0001. Line and error bars, mean ± SEM. See also Figure S4.

DDX21 binds promoters of RNAPII genes to release paused polymerase and activate transcription elongation (Calo et al., 2015). The presence of PRL3-DDX21 complexes in the nucleoplasm led us to hypothesize that PRL3 regulates transcriptional elongation via DDX21. To investigate this, we used metabolic labeling with 4-thiouridine RNA sequencing (4sU-seq) to label nascent RNA transcripts (Rabani et al., 2011) in A375 cells that stably express either *PRL3* or an empty vector control (Figure 4A). Genome-wide analysis of read coverage normalized to gene length identified an accumulation of transcripts toward the 5′ end and depletion of transcripts in the 3′ end of *PRL3-*expressing cells, and this was most pronounced for long genes (>10 kb) (Figures 4B and 4C; Table S3).

Visual inspection of individual genes supported this finding but also revealed that most of the 5′ accumulation reads arose from exons. Therefore, to ensure an analysis of nascent transcripts and to remove potential confounding effects arising from co-transcriptional splicing or low-level mRNA carry-over, we focused all subsequent analysis on the quantitation of intronic reads. We found 1,745 genes that accumulated transcripts toward the 5′ end of the gene body (5′ enriched genes) and 40 genes that accumulated transcripts at the 3′ end in *PRL3*-overexpressing melanoma cells relative to controls (Figure 4D; Table S3). Over 62% of 5′ enriched genes (1,121/1,785) showed reduced mRNA levels in *PRL3*-expressing cells compared with control (Figure 4E; Table S3). Analysis of the 5′ enriched genes revealed a striking enrichment of genes and pathways involved in endolysosomal components including late endolysosomal vesicle trafficking and/or tethering, and vacuolar ATPases required to maintain an acidic pH (Figures 4E and 4F; Table S3). Melanosome and pigment granule cellular components were also enriched in our analysis, although the genes that constitute these pathways were not specific to melanosomes, rather they were shared with lysosomes and endomembrane vesicle genes because melanosomes are lysosome-related organelles (Bowman et al., 2019; Wasmeier et al., 2008).

To determine if the PRL3-mediated repression of endolysosomal vesicle gene expression is associated with a change in cellular vesicles, we used LysoTracker Red to stain acidic vesicle compartments in live cells (Chazotte, 2011). Overexpression of PRL3 in human melanoma cells resulted in a decrease of LysoTracker-positive vesicles as measured by the total area of fluorescence per cell (Figures 4G and 4H). Of the LysoTracker-positive vesicles still present in PRL3-overexpressing cells, they were reduced for LysoTracker fluorescence but with no change in size (Figures 4I and S4A–S4C). These cellular phenotypes are reliant on the phosphatase activity of PRL3 because acidic vesicles were unchanged in cells overexpressing the phosphatase-dead PRL3^C104S^.

### PRL3 Overexpression Enriches DDX21 at the 5′ End Concomitant with Reduced Productive RNAPII Transcription

**Figure 5:**
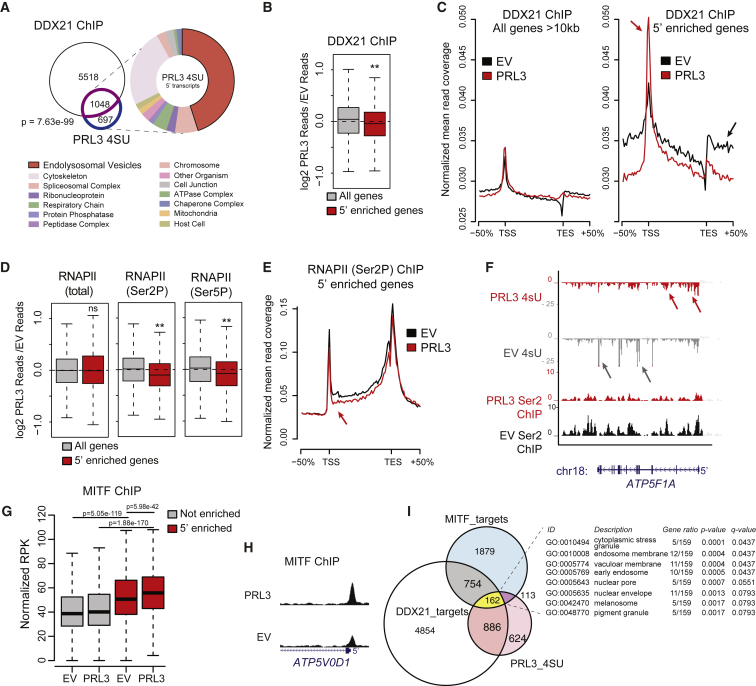
PRL3 Restrains Pol II Transcriptional Elongation of DDX21 Target Genes at MITF Targets (A) Venn diagram of overlapping genes between DDX21 ChIP targets (purple) and PRL3 5′-enriched genes (blue) (p = 7.63 e-99, Fisher's exact test). GO enrichment analysis of cellular components (false discovery rate [FDR] < 0.05). See also Table S4. (B) DDX21 ChIP-seq data as log_2_ ratios of PRL3 over-expressing (PRL3) versus empty vector (EV) control for all long (All genes) and 5'-enriched genes., ^∗∗^p = 1.61 × 10^−8^, Wilcoxon signed-rank test. (C) DDX21 ChIP-seq read profile for all long or 5′-enriched genes in control EV and PRL3-expressing cells. In 5′-enriched genes, PRL3 causes a net accumulation of DDX21 to the 5′ end (red arrows) versus EV control-treated cells (black arrow). (D) Boxplots of RNAP II (total, Ser2P, and Ser5P) ChIP-seq data shown as ratios of PRL3 versus control read depth for all long genes and 5′-enriched genes. (5′-enriched p = 0.83, 1.26 × 10^−18^ 3.93 × 10^−11^ for total, Ser2P, and Ser5P RNAP, respectively; Wilcoxon signed-rank test). (E) RNAP II (Ser2P) ChIP-seq profile for 5′-enriched genes in EV control and PRL3-expressing cells. RNAP II (Ser2P) signal is decreased at the 5′ end (red arrow) and throughout the gene body versus control cells (black). (F) 4sU RNA-seq and RNA PolII (Ser2P) ChIP-seq transcript coverage for *ATP5F1A*, an example 5′-enriched gene. In EV cells (gray) 4sU transcripts align with exons (gray arrows). In *PRL3*-expressing cells (red) transcripts are enriched at the 5′ end (red arrows) and RNAP II (Ser2P) depleted over the gene body versus EV control cells. (G) MITF ChIP-seq signal surrounding the TSS (±1 kb) for non-5′-enriched (gray) and 5′-enriched (red) genes (p values determined using paired and un-paired Wilcoxon rank sum tests for within and between gene set comparisons, respectively). (H) MITF ChIP-seq profile of *ATP5V0D1,* a 5′-enriched PRL3 target gene. Elevated MITF occupancy in *PRL3* versus control EV cells proximal to the TSS. (I) Venn diagram of DDX21 ChIP targets (white, 6,566 total), PRL3 5′-accumulated genes (pink, 1,745 total), and MITF ChIP targets (blue, 2,908 total). 162 genes overlap the three groups, highlighted in yellow (p = 6.67e−06, hypergeometric test). Table lists cellular component GO enrichment analysis of these 162 genes (FDR < 0.05). See also Table S4.

To investigate whether genes regulated by PRL3 are targeted by DDX21, we compared a DDX21 chromatin immunoprecipitation sequencing (ChIP-seq) dataset from A375 melanoma cells (Santoriello et al., 2020) to our PRL3-dependent 5′ enriched genes. We observed a significant enrichment overlap (60%; 1,048/1,745 genes; p value = 7.63e−99) between genes regulated by PRL3 and between DDX21 targets. The cellular components of these genes notably involved endolysosomal vesicle biogenesis (Figure 5A; Tables S3 and S4).

To investigate the relationship between PRL3 and DDX21, we performed DDX21 ChIP-seq in control and PRL3-overexpressing cells. PRL3 overexpression had little effect on DDX21 binding to chromatin overall but significantly reduced DDX21 binding at PRL3-regulated 5′ enriched genes (Figure 5B) as well as other known RNAPII-associated DDX21 targets (Figure S5A). Moreover, in PRL3-overexpressing cells, the distribution of DDX21 binding at 5′ enriched genes accumulated at the 5′ transcription start site (TSS) and depleted in the gene body and 3′ transcription end site (TES) (Figure 5C).

Next, we asked if the shift in DDX21 distribution at PRL3-regulated 5′ enriched genes correlated with alterations in RNAPII distribution and activity. To this end, we performed ChIP-seq for total RNAPII as well as phospho-Ser5 RNAPII and phospho-Ser2 RNAPII. The phosphorylation of the carboxy terminal domain (CTD) of RNAPII is dynamic across the length of transcribing genes. Phospho-Ser5 RNAPII is associated with transcriptional initiation, and both phospho-Ser5 and phospho-Ser2 phosphorylation are associated with elongation (Chen et al., 2018). We found that PRL3 overexpression resulted in reduced levels of both Ser5 and Ser2 forms of RNAPII at 5′ enriched genes (Figures 5D–5F). These findings suggest that PRL3 impairs DDX21 binding and distribution on chromatin, thereby reducing productive RNAPII transcription and leading to the accumulation of transcripts at the 5′ end of endomembrane vesicle genes.

### PRL3-DDX21 Transcriptional Regulation of MITF Endolysosomal Target Genes

Next, we wanted to understand how endolysosomal target genes were chosen by PRL3-DDX21. MITF has an ancestral role in regulation of the *V-ATPase* genes and a unique and selective role for regulation of genes involved in endolysosomal biogenesis (Möller et al., 2019; Bouché et al., 2016; Ploper et al., 2015; Zhang et al., 2015). To investigate whether MITF participates in the transcription of PRL3-DDX21-regulated genes, we performed ChIP-seq for total MITF using a validated antibody and confirmed the enrichment and specificity for known MITF targets (Webster et al., 2014; Figures S5B–S5D). We found an increase in MITF chromatin binding at the TSS of PRL3-regulated genes compared with all long genes (≥10 kb), with further enriched MITF binding at 5′ enriched genes in PRL3-expressing cells compared with the control (Figure 5G). Increased MITF binding at 5′ enriched genes in PRL3-expressing cells may indicate that MITF residency time is coupled to Pol II licensing and active elongation (Swift and Coruzzi, 2017). To examine these genes more closely, we performed peak-finding analysis on the MITF ChIP-seq data followed by Gene Ontology (GO) enrichment analysis for the PRL3-DDX21-MITF common target genes. We found 162 out of 1,048 (15.5%) PRL3-DDX21 target genes (Figures 5H and 5I; Table S3) had robust MITF-binding peaks and were enriched in endolysosomal factors (Figure 5I). Thus, PRL3-DDX21 transcriptional regulation is active at MITF-dependent endolysosomal genes and represents a new regulatory mechanism of MITF-controlled gene expression.

### Prl3a Negatively Regulates Endolysosomal Vesicle Accumulation in Zebrafish MSC Differentiation

**Figure 6:**
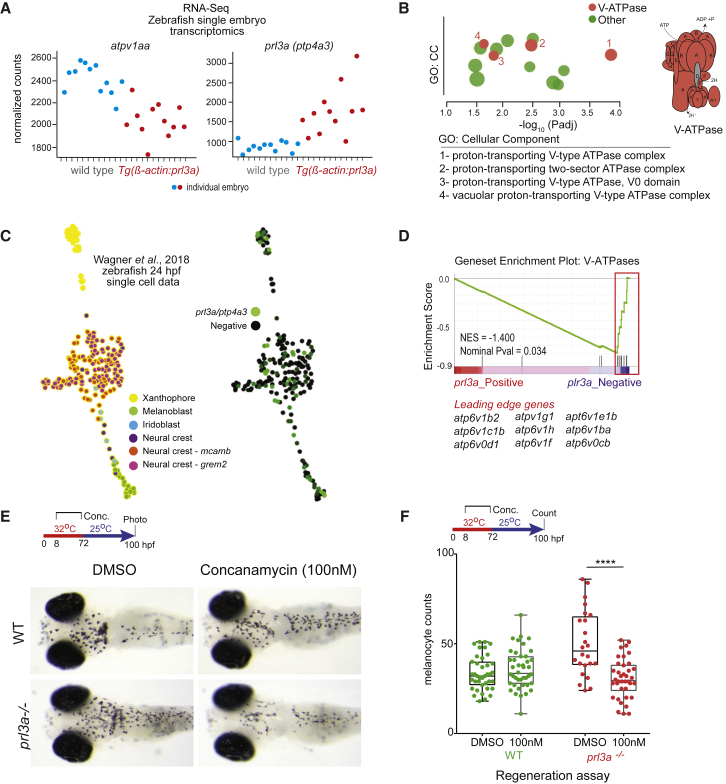
*prl3* Mutants Are Rescued by a V-ATPase Inhibitor (A) Single embryo transcriptomics of heterozygote *Tg(β-actin:prl3a)* zebrafish reveals *atpv1aa* is selectively downregulated versus wild-type control siblings. Adjusted p-value (p < 0.05) was determined by DESeq2 using the Wald test with Benjamini-Hochberg correction for multiple testing. See also Table S5. (B) g:Profiler output shows V-ATPase complex GO enrichment for *Tg(β-actin:prl3a)* differentially expressed genes versus wild-type controls. Inset: diagram of V-type ATPase, all genes encoding components labeled red are downregulated in *Tg(β-actin:prl3a)* expressing embryos. (C) K-nearest neighbor graph (SPRING webtool) of *prl3a* expression in neural crest lineages using Wagner et al. (2018) zebrafish 24 hpf single-cell data. (D) GSEA plotting enrichment of *V-ATPase* genes in the *prl3a*-negative subpopulation of pigment cells from Wagner et al. (2018). (E and F) (E) Images and (F) quantification of melanocytes in wild-type (WT) or *prl3a*^−^^/^^−^ mutant embryo ± concanamycin A in a *mitfa*^*vc7*^ regeneration assay, ANOVA using Tukey’s test, ^∗∗∗∗^p < 0.0001.

Given our findings in human cells, we hypothesized that a transcriptional mechanism underlies Prl3a function in the zebrafish MSC lineage. We performed single-embryo RNA-seq on hemizygous transgenic *Tg(β-actin: prl3a)* embryos (one copy of the transgene is not sufficient to prevent melanocyte development) and matched sibling controls (Figure 6A). We identified selective downregulation of transcripts that encode vesicle fusion, sorting, and melanosome maturation components, including almost all the components of the V-ATPase complex (Figure 6B; Table S5). Next, we analyzed zebrafish 24 hpf single-cell RNA-seq data (Wagner et al., 2018) and identified *prl3a*-expressing cells in neural crest and melanoblast populations (Figure 6C). Gene set enrichment analysis (GSEA) indicated that the *prl3a+* subpopulation was significantly depleted for *V-ATPase* transcripts, whereas *prl3a−* cells were enriched for these transcripts (Figure 6D). This is consistent with our findings in mammalian cells and with a function for Prl3a in transcriptional inhibition of these genes.

As *prl3a*- cells were characterized by increased gene expression of *V-ATPase* genes, we hypothesized V-ATPase inhibitors might restore enhanced MSC regeneration in *prl3a*^*−/−*^ mutants. To test this, we treated *prl3a*^*−/−*^ mutants with the V-ATPase inhibitor concanamycin A in a *mitfa*^*vc7*^ regeneration assay. Concanamycin A can lead to pigmentation defects at higher concentration because acidic vacuolar pH is required for melanosome formation (Dooley et al., 2013b), but when we treated embryos at low concentrations (100 nM) of concanamycin A there was no effect on wild-type melanocytes. Importantly, concanamycin A treatment during the activation of the MSC but prior to melanin synthesis reversed the regeneration phenotype in *prl3a*^*−/−*^ mutant embryos, indicating that acidic vesicles contribute to the enhanced rate of differentiation from the MSC lineage in *prl3a*^*−/−*^ mutants (Figures 6E and 6F). We did not observe an effect on wild-type regeneration possibly because the embryos already have low levels of endomembrane vesicles at this stage. These data functionally implicate V-ATPase activity in melanocyte regeneration in *prl3a* mutants.

### A *PRL3*-High Endolysosomal-Low Transcriptional Signature in Melanoma Patients

**Figure 7:**
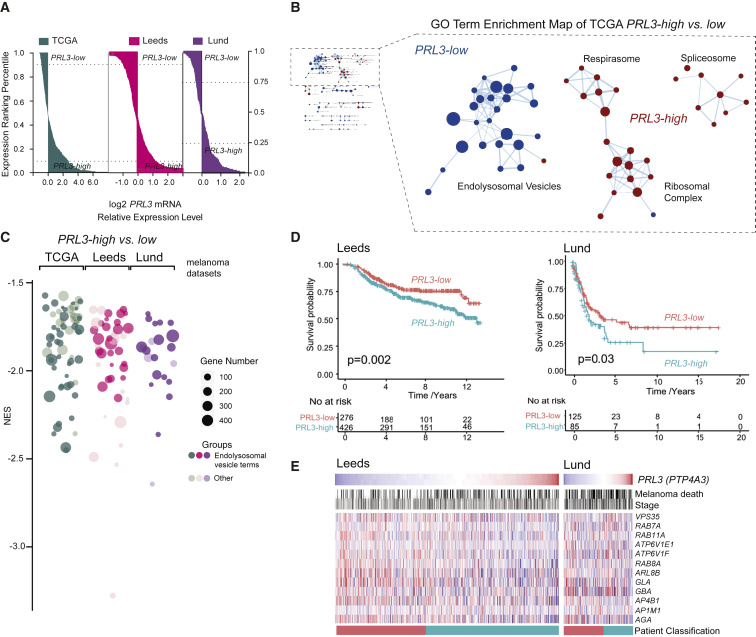
A *PRL3*-High, Endolysosomal Gene-Low Signature in Human Melanoma Samples (A) Patient samples ranked by *PRL3* RNA expression. *PRL3-high* and *PRL3-low* subgroups were defined with a 10% threshold for TCGA (n = 366) and Leeds (n = 703) and 25% threshold for the Lund dataset (stage III patients, n = 124). (B) GO enrichment analysis (cellular compartment) of *PRL3* subgroups in TCGA (Cytoscape). Node radius represents GO term gene counts. Spokes represent shared gene counts between terms. (C) Bubble plot of GO cellular components in *PRL3-high* versus*PRL3-low* melanomas. Over-representation analysis (FDR < 0.05). NES, normalized enrichment score. (D) Kaplan Meier survival curves of *PRL3-high* versus *PRL3-low* melanomas in the Leeds and Lund cohorts Leeds dataset: p = 0.002; Lund dataset: p = 0.03; logrank tests. (E) Heatmap of endolysosomal genes ranked by *PRL3* expression (mean-centered): high (red), low (blue). Melanoma deaths are indicated in black; stages are indicated by shades of gray, darker shades represent higher stages. Patient classification: *PRL3-low* (red), *PRL3-high* (green), as in (D). See also Figure S6; Table S6.

*PRL3* expression is upregulated in many cancer types and associated with increased metastasis and poor patient outcomes (Wei et al., 2018; den Hollander et al., 2016; Molleví et al., 2008); however, there is limited analysis of *PRL3* and patient outcomes in non-uveal, cutaneous melanoma. We asked if this newly identified function for PRL3 in transcriptional regulation is evident in human melanoma samples. To this end, we analyzed gene expression patterns in the melanomas with the highest and lowest *PRL3* expression from TCGA (369 metastatic patients), Leeds (703 patients; primary, treatment-naive tumors), and Lund (124 patients; stage III) cohorts (Cancer Genome Atlas Network, 2015; Cirenajwis et al., 2015; Nsengimana et al., 2015; Newton-Bishop et al., 2009, Figure 7A). Strikingly, depletion of endomembrane vesicle components was a hallmark of *PRL3*-high melanomas in all three patient cohorts (Figures 7B and 7C; Table S6). In contrast, we found stem cell-state genes enriched in *PRL3*-high melanomas (Figure S6A).

Next, to explore the impact of *PRL3* expression on patient prognosis stratification in melanoma, we performed comprehensive survival analysis to assess all possible cut points to assign patients to high or low *PRL3* expressing groups (Pearce et al., 2017). Almost half (48.6%) of the 701 possible cut points for the 702 patients in the Leeds dataset significantly associated (p < 0.05) high expression levels of *PRL3* with melanoma-specific death (Figures 7D and S6B). High *PRL3* expression was also significantly associated with melanoma-specific death in the smaller Lund dataset (Figures 7D and S6C). Consistent with our functional studies, *PRL3* expression was inversely correlated with a number of endolysosomal component genes in the Leeds and Lund melanoma patient cohorts (Figure 7E).

Notably, there was no significant association of *PRL3* with disease stage (a known prognostic factor) indicating that high *PRL3* expression is a valuable predictive marker for metastatic melanoma-specific death in all stages (Figures 7E, S6D, and S6E). This was further validated in a stage-matched sub-cohort of metastatic melanomas where high *PRL3* expression was still associated with increased disease-specific death and low *PRL3* expression with endolysosomal gene expression (Figures S6F–S6H). Thus, similar to our zebrafish and cell line models, *PRL3* expression is inversely associated with endolysosomal vesicle gene expression in clinical samples, suggesting PRL3-mediated transcriptional regulation of these genes functions in metastatic melanoma.

## Discussion

Somatic stem cells couple self-renewal with differentiation during tissue repair and regeneration, and these pathways are often dysregulated in cancer. Here, we present the conceptual advance that regulation of transcription elongation prevents premature MSC differentiation and reveal a new mechanism that regulates endolysosomal target genes via PRL3-DDX21-mediated inhibition. Given that stemness is associated with metastasis in cancer, including melanoma (Rambow et al., 2019; Oskarsson et al., 2014), we propose that Prl3a function in the MSC lineage during regeneration may be linked to its role in metastasis.

Transcription through gene bodies is subject to dynamic changes in elongation rate, and is a highly regulated process in development and differentiation (Chen et al., 2018; Jonkers and Lis, 2015; Bai et al., 2010; Maslon et al., 2019; Tan et al., 2016; White et al., 2011). We propose PRL3-DDX21 regulation of productive transcription elongation functions as a fine-tuning mechanism for matching the regenerative response with tissue needs and an opportunity for specifically targeting cancer cells in a non-regenerative setting. Phosphorylation of DDX21 is likely central to this mechanism, and our finding that the DDX21 S71A phospho-mutant reduces and restores the rate of regeneration in *prl3a* mutants points to a function for dephosphorylated Ddx21 in delaying transcription through endomembrane genes.

While it might be supposed that PRL3 regulation of MSC differentiation is via melanosome synthesis, in fact, we do not identify PRL3 5′-enriched genes that are specific to the melanosome or to melanin synthesis. Rather, the endolysosomal genes regulated by PRL3 are associated with lysosomal-related acidic organelle biosynthesis and trafficking. Notably, dysregulation of endolysosomal pathways has emerged as a hallmark of melanoma and a driver of metastasis (Alonso-Curbelo et al., 2014; Marie et al., 2020). In melanocytes and melanoma, MITF is the transcriptional regulator of *V-ATPase* gene expression (Bouché et al., 2016; Zhang et al., 2015), and these genes are required for autophagosome formation in response to starvation (Möller et al., 2019) and are also required for endolysosomal vesicles that function as concentrating centers for Wnt signaling (Ploper et al., 2015). Thus, it seems likely that PRL3-dependent restrained transcriptional elongation of endolysosomal components leads to more than simply delaying melanosome biogenesis and points to a function in maintaining a stem cell state. Indeed, the dramatic increase of early-stage melanoblasts emerging from the MSC compartment in B4-Rh-treated regenerating zebrafish embryos supports the concept that endomembrane vesicles function at an early stage of MSC-derived melanoblast development prior to pigmentation, and prior even to expression of functional Mitfa protein, as demonstrated in the *mitfa*^*vc7*^ mutants. The function of these vesicles in MSC differentiation is not known but may involve regulation of signaling pathways, such as the Notch pathway that is regulated by endomembrane vesicles and inhibits premature differentiation of the MSC in the hair follicle niche (Aubin-Houzelstein et al., 2008; Schnute et al., 2018).

PRL3 is a validated drug target for many metastatic cancers (Wei et al., 2018). A humanized antibody against PRL3 demonstrated therapeutic efficacy as monotherapy in pre-clinical models (Thura et al., 2016, 2019) and is currently being tested in a phase 1 clinical trial (ClinicalTrials.gov identifier: NCT03191682). Importantly, we find that high *PRL3* expression is an independent predictor of melanoma-specific death at all stages. Thus, all patients with *PRL3*-high melanomas may be at risk of metastasis and death and benefit from PRL3 targeted therapies.

In conclusion, despite the demonstrated function for PRL3 in metastatic cancers, an endogenous function of PRL3 activity in development has remained elusive. Our work identifies PRL3 as a lineage-specific regulator of DDX21 transcription elongation at endolysosomal genes to prevent premature MSC differentiation during regeneration. The PRL3 transcriptional elongation mechanism we present here is a new example of MITF regulatory control and of a developmental process that is co-opted by melanoma to maintain a progenitor state.

## STAR★Methods

### Key Resources Table

**Table:** 

REAGENT or RESOURCE	SOURCE	IDENTIFIER
**Antibodies**		

Rabbit anti-DDX21	Abcam	Cat # ab182156; RRID: AB_2857991
IgG protein mag Sepharose beads	Fisher Scientific	Cat # 10249574
Anti-HA-tag mAb-Magnetic Beads	MBL	Cat # M180-11
Rabbit anti-beta Tubulin	Abcam	Cat # ab6046; RRID: AB_2210370
Mouse anti-DDX21	Santa Cruz	Cat # sc-376953; RRID: AB_2819085
Rabbit Anti-HA	Abcam	Cat # ab9110; RRID: AB_307019
Mouse anti-PRL3	Santa Cruz	Cat # sc-130245; RRID: AB_2174658
IRDye 800 CW Donkey anti-rabbit IgG	LICOR	Cat # 926-32213; RRID: AB_621848
IRDye 680 RD anti-mouse secondary	LICOR	Cat # 926-68072; RRID: AB_10953628
Chicken anti-rabbit conjugated Alexa Fluor 488	Invitrogen	Cat # A21441; RRID: AB_141735
Goat anti-mouse conjugated Alexa Fluor 647	Invitrogen	Cat # A21236; RRID: AB_141725
Rabbit anti-DDX21 (ChIP grade)	Proteintech	Cat # 10528-1-AP; RRID: AB_2092705
Mouse anti-RNA-POLII (ChIP)	Novus Biologicals	Cat # C15200004; RRID: AB_2728744
Mouse anti-RNA-POLII PS2 (ChIP)	Novus Biologicals	Cat # C15200005-50; RRID: AB_2713925
Mouse anti-RNA-POLII PS5 (ChIP)	Novus Biologicals	Cat # C15200007-50; RRID: AB_2713926
Rabbit anti-MITF (ChIP)	Atlas Antibodies	Cat # HPA003259; RRID: AB_1079381
Protein G dynabeads	ThermoFisher	Cat# 10003D; 1ml

**Chemicals, Peptides, and Recombinant Proteins**		

NFN1	Maybridge	Cat # BTB05727
Enzo Life-Sciences Screen-WellTM Phosphatase Inhibitor library	Enzo Life-Sciences	Cat # BML-2834-0100
B4-Rhodanine	Sigma-Aldrich	Cat # P0108 (Sigma)
4-thiouridine	Sigma	#T4509
Biotin-HPDP	Pierce	Cat # 21341
Image-IT™ LIVE Lysosomal and Nuclear Labeling Kit	ThermoFisher	Cat # I34202
Sulforhodamine B sodium salt (SRB)	Sigma-Aldrich	Cat # S1402
Propidium iodide	Roche	Cat # 11348639001
Concanamycin A (Folimycin)	Abcam	Cat # Ab144227
AG1478	Calbiochem/Sigma-Aldrich	Cat # 658552

**Commercial Assays**		

Tol2kit gateway cloning	Kwan et al., 2007	N/A
mMESSAGE mMACHINE® T3 Kit	Ambion	Cat# AM1348
Low Input RiboMinusTM Eukaryote System v2 kit	Ambion	Cat # A15027
NEBNext Ultra Directional RNA library Prep kit for Illumina	NEB	Cat # E7420S
μMacs Streptavidin Kit	Miltenyi	Cat # 130-074-101
Illumina TruSeq Stranded mRNA Sample Prep Kit. Libraries	Illumina	Cat # RS-122-2001
NEBNext® Ultra™ II DNA Library Prep Kit for Illumina®	NEB	Cat # E7645S

**Deposited Data**		

4sU RNA-seq	This paper	GEO: GSE127855
Zebrafish single embryo transcriptomics	This paper	ENA: PRJEB12366
Gene expression of human melanoma patient samples from TCGA dataset	TCGA cBioPortal; https://www.cbioportal.org/	RRID: SCR_003193
Gene expression of human melanoma from Lund data set	https://www.ncbi.nlm.nih.gov/geo/query/acc.cgi?acc=GSE65904	GEO: GSE65904
Zebrafish single cell RNA-seq (Wagner et. al, 2018)	https://www.ncbi.nlm.nih.gov/geo/query/acc.cgi?acc=GSM3067195	GEO: GSE112294/GSM3067195
MITF, DDX21 and ChIP-seq data	This paper	GEO: GSE149929

**Experimental Models: Cell Lines**		

A375 human melanoma cell line	ATCC	ATCC: CRL-1619; RRID: CVCL_0132
Empty Vector A375 human melanoma cell line	This paper	N/A
PRL3^HA^ A375 human melanoma cell line	This paper	N/A
PRL3^HA^ C104S human melanoma cell line	This paper	N/A
C092 human melanoma cell line	Professor Nick Hayward and the ABN Cell Line Bank (QIMR Berghofer Medical Research Institute)	N/A

**Experimental Models: Organisms/Strains**		

*mitfa*^*vc7/vc7*^	Johnson et al., 2011	RRID: ZFIN_ZDB-GENO-110330-3
*nbt:dsred*	Dooley et al., 2013a	N/A
*mitfa:gfp*	Dooley et al., 2013a	N/A
*actin:prl3a;cmlc2:gfp*	This paper	N/A
*actin:prl3a;cmlc2:gfp; mitfa*^*vc7/vc7*^	This paper	N/A
*prl3aΔ20; mitfa*^*vc7/vc7*^	This paper	N/A
*prl3aΔ20; prl3bΔ14; mitfa*^*vc7/vc7*^	This paper	N/A

**Oligonucleotides**: See Table S7		

**Recombinant DNA**		

pCEP4-PRL3	Saha et al., 2001	Addgene; plasmid number #16618
pCEP4-PRL3C104S	This paper	N/A
pDONOR221	Tol2kit v1.2	plasmid #:218
p5E-actin2	Tol2kit v1.2	plasmid #:299
p3E-polyA	Tol2kit v1.2	plasmid #:302
pDestTol2CG2	Tol2kit v1.2	plasmid #:395
pCS2FA-transposase (Tol2 mRNA expression vector)	Tol2kit v1.2	plasmid #:396
TALEN nucleases	Addgene	RRID: Addgene_35992; RRID: Addgene_35993
gRNA vector, DR274	Addgene	RRID: Addgene_42250
Cas9 mRNA expression vector, MLM3613	Addgene	RRID: Addgene_42251
pSPE3-HA-RfA	Roure et al., 2007	N/A
pCS-DDX21-S71WT	This paper	N/A
pCS-DDX21-S71A	This paper	N/A

**Software and Algorithms**		

PDB2PQR 2.1.1	Dolinsky et al., 2014	N/A
MGLTools 1.5.6	Sanner, 1999	N/A
OpenBabel 2.4.1	Copyright (C) 2005-2007 Geoffrey R. Hutchison babel@geoffhutchison.net; https://pypi.org/project/openbabel/	N/A
Autodock 4.2.6	Prof. Arthur J. Olson Department of Molecular Biology, MB-5 The Scripps Research Institute La Jolla, CA 92037 USA; http://autodock.scripps.edu/downloads/autodock-registration/autodock-4-2-download-page/	RRID: SCR_012746
ZiFiT targeter	Sander et al., 2010; http://zifit.partners.org/ZiFiT/Disclaimer.aspx	N/A
FastQC (v. 0.11.3)	Babraham Institute; https://www.bioinformatics.babraham.ac.uk/projects/fastqc/	RRID: SCR_014583
STAR (v. STAR_2.5.1b)	Dobin et al., 2013	RRID: SCR_015899
RNA-SeQC (v. 1.1.8.1)	DeLuca et al., 2012	RRID: SCR_005120
Deseq2 R package (v. 1.20.0)	Love et al., 2014; https://bioconductor.org/packages/release/bioc/html/DESeq2.html	RRID: SCR_015687
RNAQC geneBody_coverage.py script (v. 2.6.4)	Wang et al., 2012	http://rseqc.sourceforge.net/
bedtools (v. v2.26.0)	Quinlan and Hall, 2010; https://github.com/arq5x/bedtools2	RRID: SCR_006646
FeatureCounts (from the subread package, v. 1.6.1)	Liao et al., 2014; http://subread.sourceforge.net/	RRID: SCR_012919
ClusterProfiler R package (v. 3.10.1)	Yu et al., 2012	RRID: SCR_016884
SPRING tools	Wagner et al., 2018	RRID: SCR_016884
scDE (v. 1.99.4)	Kharchenko et al., 2014; http://hms-dbmi.github.io/scde	RRID: SCR_016884
GSEA	Subramanian et al., 2005; http://www.broad.mit.edu/gsea/index.html	RRID: SCR_016884
g:Profiler	Reimand et al., 2007	RRID: SCR_006809
ImageJ 1.52q	National Institutes of Health, USA	RRID: SCR_003070
Prism 8 (VERSION 8.4.1) for macOS	GraphPad Software, San Diego, USA	RRID: SCR_002798

### Resource Availability

#### Lead Contact

Further information and requests for reagents should be directed to and will be fulfilled by the Lead Contact, E. Elizabeth Patton (e.patton@igmm.ed.ac.uk).

#### Materials Availability

Newly generated materials from this study are available by request from the Lead Contact, E. Elizabeth Patton (e.patton@igmm.ed.ac.uk).

#### Data and Code Availability

4sU experiments have been submitted to GEO: GSE127855. Single-embryo transcriptomics data have been submitted to EMBL-EBI ENA # PRJEB12366. ChIP-seq data have been submitted to GEO: GSE1499299. All other data supporting the findings of this study are available from the corresponding author upon reasonable request.

### Experimental Models and Subject Details

#### Zebrafish Husbandry

Zebrafish were maintained in accordance with UK Home Office regulations UK Animals (Scientific Procedures) Act 1986, amended in 2013, and European Directive 2010/63/EU under project license 70/8000 and P8F7F7E52, reviewed by the University of Edinburgh Animal Welfare and Ethical Review Body (AWERB).

#### Human Melanoma Cell Culture

A375 cells (ATCC) were cultured in DMEM and C092 were cultured in RPMI (Life Technologies) media. Both media were supplemented with 2 mM L-glutamine (Life Technologies) and 10% fetal calf serum (Life Technologies) and all cells grown at 37°C in a 5% CO_2_ humidified incubator. A375 is derived from a female patient and C092 is derived from a male patient. C092 has been authenticated through deep exome sequencing and short tandem repeat profiling.

### Method Details

#### Phenotypic Screen

Zebrafish wildtype embryos (AB, 4-5 hpf) were arrayed in a 24 multi-well plate (5 embryos/well). At 30 hpf embryos were treated with 20 μM NFN1 (Maybridge BTB05727) and co-treated with Enzo Life-Sciences Screen-WellTM Phosphatase Inhibitor library at 5, 10 and 20 μM concentrations. Embryos were assessed for NFN1 induced melanocyte ablation phenotype at 50 hpf and drugs were washed out. Melanocyte regeneration was monitored daily following washout using a Nikon SMZ 1000 stereomicroscope. While B4-Rhodanine (PRL3 inhibitor) showed the strongest regeneration phenotype, a CDC25 inhibitor (BN-82002) also showed a mild enhanced regeneration phenotype, but the melanocytes were not fully pigmented suggesting additional activity for BN-82002 and/or CDC25 in pigmentation.

#### Melanocyte Regeneration Models and Drug-Treatment of Zebrafish

For NFN1 regeneration assays, wildtype (AB) embryos were treated with 20μM NFN1 ± 20μM B4-Rhodanine for 32-50 hpf. Following washout embryos were imaged and melanocytes counted at 100 hpf, n>3 experimental replicates.

For B4-Rhodanine treatment in normal development, 4 hpf embryos were treated with B4-Rhodanine or a DMSO solvent control. Drug treatment was replenished daily until embryos were imaged at 100 hpf, n>3 experimental replicates.

For the genetic melanocyte regeneration assay, *mitfa*^*vc7*^ embryos were grown at 32°C until 48 or 72 hpf, followed by 48 or 72 hours at 25°C. Embryos were fixed between 100-120 hpf in 4% paraformaldehyde in PBS. Drug treatment of zebrafish embryos was performed from 4-5 hpf until 48 or 72 hpf as indicated. B4-Rhodanine was used at 20 μM or 10 μM (Sigma-Aldrich; Enzo Life Sciences), AG1478 was used at 6 μM (Calbiochem/ Sigma Aldrich) At 96 hpf embryos were imaged and counted, representative of 3 biological replicates. Concanamycin A (Abcam; 100nM) was added to embryos from 8-72hpf before washing out during regeneration. 120hpf regenerated embryos were collected and fixed in 4% PFA in PBST and imaged using a Leica FLIII stereomicroscope.

Adult tail fin regeneration assays were performed using a sterile razor blade to surgically remove a posterior portion of the tail in age-matched sibling fish. Tail-clipped adults were housed individually in control E3 medium or 1 μM B4-Rhodanine treatment groups. In some instances, fish were co-treated with 0.003% 1-phenyl-2-thiourea (PTU).

For lateral stripe regeneration assays, wildtype zebrafish embryos were treated 0-48 hpf with 2 μM or 6 μM AG1478 (Calbiochem/ Sigma Aldrich) and 20 μM B4-Rhodanine. Embryos were raised at 32°C. For a subset of experiments embryos were raised at 28°C and treatment was continued from 48-96 hpf using 3 μM AG1478 and 10 μM B4-Rhodanine. At 96 hpf embryos were exposed to a bright light before imaging/counting to contract melanocytes. Representative of 3 biological replicates.

#### Imaging *Tg(mitfa:GFP; nbt:dsRED)* Transgenic Zebrafish

Embryos at 4 hpf *Tg(mitfa:GFP;nbt:dsRED)* were arrayed in 6-well plates (Corning) containing 0.1% DMSO, 20 μM B4-Rhodanine (Sigma-Aldrich), 8 μM of NFN1 (Maybridge BTB05727) or both 20 μM B4-Rhodanine and 8 μM of NFN1 in 3 ml of E3 embryo medium. DMSO and B4-Rhodanine treatments were performed between 4 and 48 hpf; NFN1 treatment was performed between 32 and 50 hpf. An alternate treatment regime included 0.1% DMSO, 20 μM B4-Rhodanine, 20 μM of NFN1 or a combination of B4-Rhodanine and NFN1 from 30-70 hpf. Images of randomly picked embryos were acquired using a Leica SP5 confocal (Leica Microsystems) using LAS-AF software. Images were scanned using a HCX PL Fluotar 20X/0.5 objective and zoom = 5. Data were analyzed using Fiji 1.0 and 64bit Java8. Representative of 3 biological repeats.

#### Whole Mount *In Situ* Hybridization

B4-Rhodanine treated (20 μM) *mitfa*^*vc7*^ embryos grown at 32°C for 48 hours were fixed in 4% paraformaldehyde and dehydrated in 100% methanol until processed. The whole mount *in situ* hybridization protocol used was adapted from https://wiki.zfin.org/display/prot/Whole-Mount+In+Situ+Hybridization. Riboprobes for *mitfa*, *sox10* and *foxd3* were kindly provided by R.N. Kelsh (University of Bath, UK). Representative of 2 biological replicates.

#### *In Silico* Modelling of PRL3 with B4-Rhodanine

Water molecules and other heteroatoms were removed from the NMR structure of human PRL3 (PDB 1V3A) and the program PDB2PQR 2.1.1 used to assigned position-optimized hydrogen atoms, utilizing the additional PropKa algorithm with a pH of 7.4 to predict protonation states. The MGLTools 1.5.6 utility prepare_receptor4.py was used to assign Gasteiger charges to atoms. Hydrogen atoms were assigned to compound structures using OpenBabel 2.4.1, utilizing the -p option to predict the protonation states of functional groups at pH 7.4. The MGLTools utility prepare_ligand4.py was used to assign Gasteiger charges and rotatable bonds. Autodock 4.2.6 was used to automatically dock the compounds into the phosphate-binding pocket of the crystal structure. A grid box that encompassed the maximum dimensions of the cognate ligand plus 12 Å in each direction was used. The starting translation and orientation of the ligand and the torsion angles of all rotatable bonds were set to random. The Autogrid grid point spacing was set at 0.2 Å. The Autodock parameter file specified 50 Lamarckian genetic algorithm runs, 7,625,700 energy evaluations and a population size of 300. The lowest energy conformation of the most populous cluster was predicted to bind with a Ki of 878 nM (± 2.5 kcal/mol).

#### Generation of Zebrafish Transgenic and Mutant Lines

*Tg(ß-actin:prl3a)* transgenics were generated by first cloning zebrafish *prl3a* cDNA, amplified from wild type zebrafish total RNA, using RT-PCR with primer sequence; forward: 5’-GGGGACAAGTTTGTACAAAAAAGCAGGCTATGGCTCGCATGAACCGACC-3’ and reverse:5’-GGGGACCACTTTGTACAAGAAAGCTGGGTTCACATGATACAGCACTTGTTC-3’.

The *ß-actin:prl3a* targeting vector was generated using the Tol2kit gateway cloning method 38.The prl3a PCR product was cloned into pDONOR221, producing a middle entry vector, pMEprl3a.pME-prl3a was cloned with the zebrafish actin promoter from p5E-actin2 (Tol2kit v1.2,plasmid #:299), and the SV40 polyA sequence from p3E-polyA into the pDestTol2CG2 destination vector.

The *Tg(ß-actin:prl3a)* targeting vector was mixed with Tol2 mRNA and microinjected into 1-cell stage wild type embryos, at a final concentration 25 ng/μl and 35 ng/μl respectively. Zebrafish embryos expressing green fluorescent protein (GFP) transgenic marker in cardiac tissue were selected and grown to adulthood before crossing with wildtype zebrafish to obtain the F1 generation with cardiac GFP expression. The prl3a mutant line was generated using TALEN nucleases. prl3a exon 7 (ENSDARG00000039997) was targeted with a TALEN pair that were kindly provided by Keith Joung (Addgene, plasmid # 35992 and 35993). The target sequence used was; TCTCCTGAAAAGGCGCTTcatcgaggagcccggcTGCTGCGTGGCTGTGCA. The TALEN binding site is written in upper case, and spacer sequence in lower case. A 273 bp sequence surrounding the target site was amplified using the following primers; forward: 5’-CTATGGCAGTCGATTGCTTTGG-3’ and reverse: 5’-CTAAGTACTAAGTGGTTGGTCG-3’. A F1 fish carrying a mutation of 20 bp deletion (GAGGAGCCCGGCTGCTGCGT) at the target site was selected and named *prl3aΔ20* for further studies. The *prl3bΔ14* mutant line was generated using CRISPR/Cas9 technology. A guide RNA (gRNA) target was identified in exon 4 at the prl3b locus (ENSDARG00000054814) using the webbased ZiFiT targeter software (http://zifit.partners.org/). The target sequence was 5’- GGTGGCTGTGGCTCTTATAGagg-3’ (PAM sequence in lower case). Guide RNA expression vectors were constructed by in vitro synthesis of gRNA and Cas9 mRNA. The gRNA vector, DR274 and Cas9 mRNA expression vector, MLM3613 were kindly provided by Keith Joung (Addgene plasmid # 42250 and 42251). Screening identified the founder prl3bD14, which contained a 14 bp deletion (5’- TGGCTCTTATAGAG-3’) at the target site.

#### Zebrafish Morpholino Oligonucleotides and RNA Injections

For the *prl3* morpholino, 11ng fluorescein-labeled morpholino oligonucleotide was injected (5’- ATAGTTGTGCTTCCTTCCGACTCAA-3’ or 5’-GACCGTTCAACCAGGCCATACTGGA-3’) (Gene Tools, LLC) as well as the random control. This morpholino blocked expression of *prl3a* and *prl3b*. Regenerating melanocytes in *prl3* morpholino-injected and control-injected fish were tested in the *mitfa*^*vc7*^ regeneration assays with embryos raised at 32°C until 54 hpf before down-shifting embryos to 25°C and quantification and imaging of regenerating melanocytes at 96 hpf. Representative of 3 biological replicates.

Embryos were injected at the one-cell stage with a 2.5 ng fluorescein-labeled morpholino against ddx21; (5’-ATTCTGGGAGACTCTTTCACGGCAT-3’) and fluorescein scrambled control morpholino (5’ATTgTcGGAAcACTgTTTcACGGCAT-3’) (Gene Tools, LLC). Regenerating melanocytes in *ddx21* morpholino-injected and control-injected fish were tested in the *mitfa*^*vc7*^ regeneration assays with embryos raised at 32°C until 72 hpf before down-shifting embryos to 25°C and quantification and imaging of regenerating melanocytes at 100 hpf. Representative of 3 biological replicates.

The *prl3a* RNA expression plasmids were constructed by gateway cloning. The *prl3a* phosphatase mutant C104S was generated from the middle entry vector pME-prl3a with a pair of mutagenesis primers: Forward CGTGGCTGTGCACTCCGTGGCCGGTTTAGGAAGAGC, Reverse GCTCTTCCTAAACCGGCCACGGAGTGCACAGCCACG. Both pME-prl3a and pMEprl3a C104S were assembled with the destination vector pSPE3-HA-RfA by performing a single-site LR reaction. The resultant vector pSPE3-prl3a and pSPE3-prl3a C104S were linearized with SfiI (NEB) and used as template for in vitro synthesis of wildtype and mutant *prl3a* mRNA. All mRNA was transcribed with mMESSAGE mMACHINE® T3 Kit (Ambion). To overexpress prl3a mRNA, 2 nl of mRNA (300 ng/μl) was injected into each 1-cell-stage embryos.

Wild type human *DDX21* was cloned into pCS2 at the BamH1 /Xho1 restriction enzyme sites. S71A was generated by direct mutagenesis using the Q5 Site-Directed Mutagenesis kit, following manufacturer’s instructions. *DDX21* and *DDX21(S71A)* mRNA (200 pg) were injected into 1-cell-stage *mitfa*^*vc7*^ embryos, maintained at 32°C for three days, and shifted to room temperature, at which time the embryos were photographed for melanocyte count analysis. Zebrafish embryos were dose sensitive to DDX21 or the phospho-site mutant DDX21 S71A such that higher concentrations lead to developmental delay and toxicity. However, lower doses were well tolerated. Representative of 3 biological replicates.

#### Mass Spectrometry

N-terminus tagged GST-Prl3a recombinant protein was expressed, purified and resuspended in 20 mM Imidazole NP40 lysis buffer (Thermofisher) and sonicated for five seconds. The samples were pre-cleared using sepharose beads (Sigma), centrifuged multiple times and then mixed with Dynabeads (ThermoFisher). Zebrafish embryos were lysed in RIPA buffer (Sigma) containing cOmplete ULTRA and PhosSTOP tablets (Roche) and ground for 2 minutes using an automatic motor pestle. The samples were centrifuged, and supernatant transferred into a new tube. The GST-Prl3a WT and Prl3aC104S bound proteins were incubated with zebrafish embryo lysate for 2 hours. GST-Prl3 pulldowns were digested, analyzed and quantified as previously described 39. Endogenous DDX21 was immunoprecipitated from 500 μl human A375 cell lysates using rabbit anti-DDX21 antibody (Abcam), which was added to the total cell lysate. The protein antibodies-IgG protein agarose beads (GE Healthcare) were used. The immunoprecipitated protein were on-bead digested with trypsin on a KingFisher Duo robotic workstation. Peptides were analyzed on a Lumos Fusion mass spectrometer with a 1s cycle-time, 120K resolution in MS and HCD-MS/MS in the ion trap. Raw files were processed and analyzed by MaxQuant. DDX21 phosphorylation sites were quantified by normalizing the peptide intensity over DDX21 intensity. 0-values were set to not-a-number (NaN) if the peptide was identified in one of the replicates or set to 0 if not detected. Data table represents the median intensity, error bars are S.E.M., Students t-test, N was 6 for empty vector control, EV and 10 for PRL3^HA^ overexpression.

#### PRL3 Stable Transfection

HA-tagged PRL3 (Addgene) and PRL3-C104S plasmids were linearized and transfected with Attractene transfection reagent according to manufacturer's instructions (Qiagen). Targeted A375 cells were maintained in hygromycin containing media at concentration of 100 μg/ml. PRL3^HA-C104S^ was generated by site-directed mutagenesis of wild type PRL3 in pCEP4-PRL3 plasmid with the following primers sequences; forward: 5’-GCGTGGCTGTGCACTCCGTGGCGG-3’ reverse: 5’- CCGCCACGGAGTGCACAGCCACGC-3’.

#### Co-IP and Western Blotting

Empty vector and PRL3^HA^ cultured A375 cells were washed in ice cold PBS and lysed in RIPA buffer (Sigma) supplemented with cOmplete ULTRA and PhosSTOP tablets (Roche). The lysate was centrifuged at high speed to remove cell debris and the supernatant transferred into a new tube. Protein concentration was measured using Pierceprotein detection kit (Pierce) and measured on a Nanodrop. For PRL3 protein, pull-down anti-HA-tag mouse monoclonal magnetic agarose beads (MBL) were used. Membranes were blocked with 2% BSA (Sigma) and incubated with rabbit anti-beta Tubulin (Abcam), rabbit anti-DDX21 (Abcam), mouse anti-DDX21(Santa Cruz), rabbit anti-HA (Abcam), mouse anti-PRL3 (Santa Cruz) and 1:1000 rabbit anti-PRL3 (Abcam) antibodies. The primary antibodies were detected with either anti-rabbit or anti-mouse LICOR fluorescent antibodies (LICOR) and scanned using the Odyssey imaging instrumentation.

#### Fluorescent Immunostaining and Lysotracker

C092 cells were kindly provided by Professor Nick Hayward and the ABN Cell Line Bank (QIMR Berghofer Medical Research Institute). Cells were cultured on high precision cover-glass (Zeiss, Germany) in 6-well plates and fixed in 4% paraformaldehyde for 5 minutes at room temperature. Fixed cells were washed three times for 5 minutes in TBST and blocked in 3% BSA in TBST for 1 hour. Fixed cells were incubated with primary antibodies, rabbit anti-DDX21 (Abcam) and mouse anti-PRL3 (Santa Cruz), overnight at 4°C. The next day, the cells were washed three times in PBS and incubated with anti-rabbit conjugated Alexa Fluor 488 and anti-mouse conjugated Alexa Fluor 647 antibodies (Invitrogen). Fixed cells were washed, nuclear stained with DAPI (Sigma) and mounted in Vectashield® mounting media.

Super-resolution images were acquired using Structured illumination microscopy. 3D SIM images were acquired on a N-SIM (Nikon Instruments) using a 100x 1.49NA lens and refractive index matched immersion oil (Nikon Instruments). Samples were imaged using a Nikon Plan Apo TIRF objective (NA 1.49, oil immersion) and an Andor DU-897X-5254 camera using 405, 488 and 640nm laser lines. Z-step size for Z stacks was set to 0.120 μm as recommended by manufacturer's software. For each focal plane, 15 images (5 phases, 3 angles) were captured with the NIS-Elements software. SIM image processing, reconstruction and analysis were carried out using the N-SIM module of the NIS-Element Advanced Research software. Settings for acquisition and reconstruction were identical in all images.

Cells expressing empty vector, PRL3^HA^ and PRL3^C104S^ were cultured in glass plates (In Vitro Scientific). The cells were stained with Lysotracker Red (ThermoFisher) for an hour in phenol red free media. Images of the cells were captured with a Leica SP5 confocal (Leica Microsystems) using the LAS-AF software. Representative of 2 biological replicates.

#### Cell Cycle Analysis

Empty vector and PRL3^HA^ cells were harvested and washed in PBS-buffer before being fixed in cold 70% ethanol for 30 minutes. Cells were centrifuged and washed twice in PBS-buffer. To ensure that only DNA was stained, the cells were treated with 5 μg/ml RNAse A and stained with 10 μg/mol Propidium Iodine solution (Roche). The samples were analysed on a LSR-Fortessa (BD Biosciences UK) and cell cycle graphs were generated using the FlowJo™ software.

#### SRB-Assay

Growth curves for empty vector and PRL3^HA^ cells were determined by using the sulforhodamine-B (SRB) assay (Sigma-Aldrich). 2000 cells per well was plated in 96-well plates and fixed at day 1, 2 and 3 post plating in 10% trichloroacetic acid for 1 hour at room temperature. The cells were then stained with 0.4% SRB for 30 minutes at room temperature. Plates were washed and analysed by dissolving the SRB stained cells in 10mM Tris-buffer and the absorbance measured at 564 nm.

#### 4sU RNA Labelling, cDNA Library Generation and Sequencing

A375 cells stably expressing empty vector or PRL3^HA^ at 80% confluency were treated with 500 μM 4-thiouridine (Sigma) and incubated for 20 min at 37°C in a 5% CO_2_ humidified incubator. After 20 minutes, the media was removed, and the cells were immediately lysed using Trizol ™ (Invitrogen). The samples were incubated for 5 minutes at room temperature to allow nucleoprotein complexes to dissolve. After Trizol incubation, chloroform was added, and the samples were vigorously shaken for 15 seconds by hand followed by incubation at room temperature for 2-3 minutes. Samples were then centrifuged at 13,000 rpm for 15 minutes at 4°C and the upper aqueous layer transferred to a new tube. RNA was precipitated with isopropanol. Samples were vortexed briefly and incubated for 10 min at room temperature before centrifugation. The supernatant was removed, and samples were washed with 80% ethanol, vortexed briefly and centrifuged at 13,000 rpm for 10 minutes at 4°C. RNA was resuspended in water and dissolved by heating the RNA samples at 40°C for 10 minutes. Any potential contaminating DNA was removed by using a TURBO DNA-free kit (Ambion) following manufacturer’s instructions. RNA was then transferred to a QIAshredder column and centrifuged for 1 minute. Flow through, containing RNA, was transferred to a fresh tube and RNA concentration determined. Roughly 100 μg RNA per sample was biotin labeled using biotin-HPDP (1 μg per sample) (Pierce) in biotinylation buffer (100 mM Tris pH 7.5, 10mM EDTA) and incubated for 1.5 h at room temperature with movement. Unincorporated biotin-HPDP was removed by transfer to a Phase Lock Gel heavy tubes (Eppendorf) and washed with an equal volume of chloroform. Samples were incubated for 2-4 minutes and centrifuged for 5 min at 4°C. The upper phase was transferred into new Phase Lock Gel heavy tubes and the chloroform step repeated. Samples were transferred into new tubes and RNA was precipitated by adding 1/10 volume of 5M NaCl and equal volume of isopropanol. Tubes were inverted to mix and incubated at room temperature for 10 minutes and centrifuged for 20 min at room temperature. The RNA was washed in 80% ethanol and resuspended in water. To clean the biotinylated RNA, biotinylated 4sU labeled RNA was mixed with streptavidin beads and incubated for 15 minutes at room temperature. The μMACS columns (Miltenyi) were equilibrated prior to use. The columns were placed on a magnetic stand and washed with washing buffer (100 mM Tris pH 7.4, 10 mM EDTA, 1M NaCl and 0.1% Tween 20) at room temperature. Freshly prepared elution buffer (100mM DTT) and RNA-bead mix was added to the μMacs columns and the flow though was discarded. Columns were washed with 65°C pre-heated washing buffer 3 times and then 3 times with room temperature washing buffer. RNA was eluted from the columns using elution buffer containing 100 mM DTT. Flow-through was collected in a tube containing RLT buffer (RNeasy MinElute Cleanup kit). The RNA flow-though was precipitated with 100% ethanol. Samples were cleaned up using RNAeasy MinElute Spin Columns according to manufacturer’s instructions. The RNA was cleaned from contaminating cytoplasmic and mitochondrial ribosomal RNA by using a Low Input RiboMinusTM Eukaryote System v2 kit (Ambion), following manufacturer’s instructions. The ribosomic RNA-depleted RNA was quantified using Qubit®3.0 fluorometer and Qubit RNA HS Assay kit (Thermo). cDNA libraries were prepared using NEBNext Ultra Directional RNA library Prep kit for Illumina (NEB) following manufacturer’s instructions. NEBNext® Multiplex Oligos for Illumina® NEBNext® Multiplex Oligos for Illumina® (Index Primers Set 1) were used to barcode the RNA replicates. Barcoded cDNA libraries were mixed together and sequenced by Edinburgh Genomics (University of Edinburgh) using HiSeq 4000 150PE to yield at least 290M + 290M reads per lane.

#### RNAseq Pipeline

Raw FASTQ sequence reads were quality checked using FastQC (v. 0.11.3) and aligned to the human genome (GrCh38) assembly using STAR (v. STAR_2.5.1b) software using the default parameters. The quality of the resulting alignment to the transcriptome (Ensembl annotation version GRCh38.91) was also checked using RNASeqQC (v. 1.1.8.1). Raw counts of reads covering the transcriptome (Ensembl annotation version GRCh38.91) were obtained using htseq-count (0.6.1) with the “-s reverse” option. Differential expression was analyzed using the Deseq2 R package (v. 1.20.0).

#### Genome Wide Elongation Estimate

For exonic regions, the RNAQC geneBody_coverage.py script (v. 2.6.4) was used with BAM alignment files and genome annotation in bed format (Ensembl annotation version GRCh38.79, downloaded from the RNAQC website) to obtain a genome wide profile graph (or a subset of genes longer than 25kb and with more than 20 exons “long genes”, or a subset of genes smaller than 2kb “short genes”). Elongation estimate calculation per gene Intronic regions were used for this analysis. Reads were counted on the first and 4th quarter of the gene length. To prepare and select the intronic regions, all exons positions were downloaded from Ensembl. These were merged using bedtools (v. v2.26.0) to create one set of pseudo-exons per gene and the corresponding pseudo-introns positions. Overlapping genes were removed from the analysis, including non-coding transcripts and small RNAs. The intronic positions were extracted from the corresponding first and fourth quartile of each gene. FeatureCounts (from the subread package, v. 1.6.1) was used to count reads over the positions and we normalized the raw counts with the size of the intronic regions used for respective gene. The ratio calculation was generated by removing zero counts, which may be due to genuine low expression, questionable gene model or unused isoforms and lack of intronic sequence in the respective quarter. The 5’ normalized intronic counts were divided by the normalized 3’ intronic counts. The ratios produced were statistically analyzed by simple independent two group Student’s t-test for 3 replicates. (https://stat.ethz.ch/R-manual/Rdevel/library/stats/html/t.test.html Welch Two Sample t-test, two.sided). FDR was calculated by multiple comparison correction using BH methods (https://stat.ethz.ch/R-manual/Rdevel/library/stats/html/p.adjust.html).

#### Whole-Cell ChIP of A375 Cells

Empty vector and PRL3 stable overexpressing cells were cultured to 80% confluency and harvested by dissociation with trypsin in PBS/EDTA. Cells were resuspended in PBS and immediately fixed in 1% formaldehyde in PBS for 10 minutes at room temperature. The fixation was neutralised by the addition of glycine and incubated for an additional 5 minutes. The cells were washed in cold PBS. Cell pellets were resuspended in 150 μl cold lysis buffer (1% SDS, 10mM EDTA, 50 mM Tris-HCl pH8.1, 1x protease inhibitor cocktail, 1x PhosSTOP phosphatase inhibitors (Roche) and fresh 1mM DTT) and supplemented with 1350 μl 1% Triton X IP dilution buffer (1% Triton X, 20mM Tris-HCl pH8.1, 150mM NaCl, 2mM EDTA, 1x protease inhibitor cocktail (Roche), 1x PhosSTOP phosphatase inhibitors (Roche) and fresh 1mM DTT and 1mM PMSF) and incubated on ice for 10 min.

Lysed cells were sonicated on ice for 5x30 seconds with a probe sonicator followed by 50-60 cycles (30 sec on / 30 sec off) in a chilled bioruptor (Diagenode) to yield chromatin fragments ranging between 200 – 800 bp in length. For MITF ChIP, lysed cells were sonicated in a chilled SoniPrep150 probe sonicator for 15x30 seconds with a 30 seconds gap between burst. Sheared chromatin was centrifuged at max speed for 10 minutes at 4°C and the soluble supernatant transferred to new tubes. 500 μl chromatin samples were supplemented with 5 μl (5mg/ml) BSA.

10% of the input was stored and the rest was used for the immunoprecipitation. Anti-DDX21 (Proteintech; cat no. 10528-1-AP), RNA-POLII tot, RNA-POLII PS2, PS5 (Novus Biologicals) and MITF (Atlas Antibodies) were pre-bound to proteinG dynabeads (Thermo Fisher) in 10% w/v BSA in PBS according to the manufacturer’s instructions (Life Technologies) for approximately 1 h at 4°C with rotation following which free antibody was removed with 3 washes of cold 10% w/v BSA in PBS. Chromatin and proteinG beads were combined and incubated over night at 4°C (at a ratio of 500 ng of bead bound antibody per 1M cell equivalents of chromatin). Samples were washes at 4°C with rotation through the following series: 2 times in 1% Triton X IP dilution buffer, 2 times with ChIP wash A (50mM HEPES ph7.9, 500mM NaCl, 1mM EDTA, 1% Triton X-100, 0.1% Na-deoxycholate, 0.1% SDS. 1x protease inhibitor cocktail, 1x PhosSTOP phosphatase inhibitors (Roche) and fresh 1mM DTT) and 2 times with ChIP wash B (20mM Tris pH 8.0, 1mM EDTA, 250mM LiCl, 1% NP-40, 0.1% Na-deoxycholate, 1x protease inhibitor cocktail, 1x PhosSTOP phosphatase inhibitors (Roche) and fresh 1mM DTT). Finally, the samples were washed with TE (1mM EDTA, 10mM Tris pH8.0). The samples were resuspended in TE and supplemented with preheated 37°C Extraction buffer (0.1M NaHCO_3_ and 1%SDS), vortexed and incubated for 15 minutes at 37°C on a vibrating platform.

The pH of the extracted chromatin was adjusted by adding 6μl 2M Tris-HCl pH6.8 following which both the ChIP and input samples were incubated with 20μg RNAseA at 65°C for 1 hour. Cross-links were reversed and the protein degraded by the addition of 20μg Proteinase K and incubation at 65°C for 6-8 h. Following removal of the dynabeads from the ChIP samples, DNA was purified using a Qiagen PCR cleanup kit following manufacturer’s instructions. DNA libraries were prepared using NEBNext® Ultra™ II DNA Library Prep Kit for Illumina® kit and NEBNext® Multiplex Oligos for Illumina® NEBNext® Multiplex Oligos for Illumina® (Index Primers Set 1) following manufacturer’s instructions.

#### MITF ChIP qPCR

ChIP-MITF DNA and input were used for qPCR using Sybr Green master Mix (Roche) with added primers for *MLNA* (for 5’-TGG GTT CTT CCA ATG TGT CA-3’; rev 5’-TTT ATG CAT GGT CAC GTG GT-3’), *ATPG6V1G1* (for 5’-TCC TCT CTT GAC GTT GAG CA-3’; rev 5’-CTA CCC TGT CGC TGG TTC AC-3’), *ATPG6V1E1* (for 5’-GTA AAG GAA CCC GAG ATC TGC-3’; rev 5’-CAA TGC TAG GCC GGT GAA C-3’), *FAM129A* (for 5’-CCT CTT GCC TCC TGT CTC TC-3’; rev 5’-CTT GCC CTC GTC CAG CTG-3’), negative controls *ACTB* (for 5’-CTG GGT TTT ATA GGG CGC CG-3’; rev 5’-GCC GTT CCG AAA GTT GCC TT-3’) and *ACTB* intergenic region (for 5’-CCA CAA AAG ACT GAA GAC ACG G-3’; rev 5’-ACT TGT TCC TGT GCA CTA TGG T-3’′). Samples were run on a Lightcycler480.

#### ChIP-seq Mapping and Analysis

ChIP-seq data was mapped to the human genome (GRCh38 build) using bowtie2 with default options for single end sequencing to generate SAM files. Using the HOMER package, SAM files were converted into tag directories and multi-mapping reads were removed using makeTagDirectory -unique -fragLength 150. Mapped regions, that due to fragment processing, extended beyond the end of the chromosomes were removed using removeOutOfBoundsReads.pl with chromosome lengths for GRCh38. Replicate data, was combined at this stage and genome browser files (.bw) were generated using makeUCSCfile with the following options: -bigWig -fsize 1e20 -strand both -norm 10e7. The data processing and generation of signal profile plots were performed as described for the 4sU-seq data.

#### Zebrafish Single-Embryo RNA-Seq

Total nucleic acid was isolated from single embryo hemizygous transgenic prl3a and sibling embryos at 50 hpf. Total nucleic acid was treated with DNAseI (NEB, Catalogue number M0303L) and 12 replicates per genotype were processed. Ambion ERCC spike-in mix 2 (Cat. No. 4456740) was added to 200 ng RNA according to the manufacturer’s instructions and sequencing libraries were prepared using the Illumina TruSeq Stranded mRNA Sample Prep Kit. Libraries were pooled and sequenced on Illumina HiSeq 2500 in 75 bp paired-end mode. Sequencing data were assessed using FastQC and aligned to the GRCz10 reference genome and Ensembl 86 transcriptome using TopHat2. Read counts per gene were generated using htseq-count and used as input for pairwise differential expression analysis using DESeq2. All sequences are placed at ENA under the following ENA IDs: ERS1447344, ERS1447360, ERS1447337, ERS1447351, ERS1447356, ERS1447361, ERS1447362, ERS1447330, ERS1447346, ERS1447366, ERS1447352, ERS1447358, ERS1447329, ERS1447353, ERS1447327, ERS1447339, ERS1447338, ERS1447342, ERS1447341, ERS1447363, ERS1447368, ERS1447334, ERS1447355, ERS1447348. GO enrichment analysis was carried out at: https://biit.cs.ut.ee/gprofiler/gost under default settings with the added option of “Ordered query”.

#### Zebrafish Single Cell RNA-seq Analysis

Single cell RNA-seq data at 24 hpf was downloaded from the GEO database with accession number GEO - NCBIGSE112294/GSM3067195 (https://www.ncbi.nlm.nih.gov/geo/query/acc.cgi?acc=GSM3067195) and uploaded to SPRING (https://kleintools.hms.harvard.edu/tools/spring.html) for clustering, analysis and generation of k-nearest-neighbor graph following online instructions. The k-nearest neighbor SPRING plot was generated using 207 cells and 1092 genes using default settings. The same raw data (GEO-NCBI:GSM3067195_24hpf) was used to perform the GSEA analysis. Raw counts for cells belonging to clusters 139 (Neural Crest), 182 (Xanthoblasts), 183 (Melanoblasts), 184 (Iridoblasts) were extracted and sorted by prl3a expression with the following parameters (prl3a negative, log_2_ prl3a counts <1; prl3a-positive, log_2_ prl3a counts ≥1). Differential expression between prl3a-positive cells and prl3a-negative cells was performed using the scDE R package (v. 1.99.4) and the obtained list (ranked by Z-score) was used to check enrichment of VATPases genes using GSEA, http://www.broad.mit.edu/gsea/index.html. The V-ATPase genes query geneset was obtained from the zebrafish single-embryo RNA-Seq data.

#### Global Analysis of Human Melanoma Patient Samples

The gene expression and prognosis of human melanoma patient samples used were downloaded from TCGA cBioPortal (https://www.cbioportal.org/); from GEO database for the Lund data set, GEO-NCBI GSE65904 (https://www.ncbi.nlm.nih.gov/geo/query/acc.cgi?acc=GSE65904); and from European Genome-phenome Archive (EGA) database for the Leeds Melanoma Cohort (LMC) gene expression study (EGA: EGAD00010001561). Patient samples were ranked based on the PRL3 expression level. PRL3^High^ and PRL3^Low^ subgroups were defined with a 10% threshold for TCGA (n=366) and Leeds (703) and 25% threshold for the Lund dataset (stage III patients, n=124). For TCGA data, the whole transcriptomic expression values of *PRL3-high* and *PRL3-low* subgroups were retrieved from Genomic Data Commons (GDC, https://portal.gdc.cancer.gov). Comprehensive survival analysis of publicly available melanoma gene expression datasets were analysed using the SurvivALL R package (Pearce et al., 2017). The *PRL3-low/PRL3-high* groups were determined by assessing all possible cut-points using the survivALL R package, the cut-points with the lowest p-value are shown (Figure S6). High *PRL3* was not associated with outcome in the TCGA melanoma cohort, likely due to heterogeneity of patients, drug treatments and follow-up information limited to overall survival in this cohort (Liu et al., 2018).

### Quantification and Statistical Analysis

Statistical details of the experiments, n numbers, and dispersion and precision measurements can be found in the figure legends.

Counts of dorsal melanocytes in the head and trunk region were performed either blinded or using the Cell Counter plugin on ImageJ Fiji. Statistics for regeneration assays were performed using GraphPad Prism 8. For all regeneration assays, a normal distribution and equal variance were assumed. For assays with more than two groups, data was analyzed through Analysis of variance (ANOVA), using Tukey’s multiple comparison test. For assays with two groups, an unpaired two-tailed Student’s t-test was used. For regeneration assay box plots: boxes represent 25^th^ to 75^th^ percentiles, lines are plotted at median. Whiskers represent Min to Max.

For statistical analysis of the ChIP-seq data, read depths were quantified across all genes (whole gene body; ENSEMBL hs_GRCh38_p12) using the ‘annotatePeaks.pl’ function in the HOMER suite with the following parameters. -size "given" -len 0 -strand both -norm 10e7. All genes equal to, or greater than 10 kb in length were normalised across samples (per ChIP - e.g. DDX21) using the ‘normalizeQuantiles’ function in the limma R package. All subsequent statistical tests and plots (boxplots) were performed in R.

Statistical Gene Ontology analysis: The ClusterProfiler R package (v. 3.10.1) with associated R packages was used to generate biological term classification and gene enrichment analysis as described (Yu et al., 2012). The software Cytoscape was used to identify the predominant GO-term clusters from the gene ontology analysis results (Shannon et al., 2003).

Lysotracker image analysis was done using ImageJ particle analysis. Optimal imaging conditions were selected using nuclei staining (particle analysis, threshold 84/255, >25 μm^2^ size). Lysotracker staining was determined by particle analysis (threshold 215/255, 0-infinity μm^2^ size, 0-1 circularity) in each cell, and lysotracker staining intensity was determined from the original image. Total area of all particles analysed per cells was plotted, and proportional intensity per cell was calculated using the formula: SUM(M^∗^A)/SUM(A), where M is mean intensity per particle and A is area (μm^2^) of each particle. Width of lysotracker-positive particles were calculated using ImageJ, plot profile function, focusing on cross-sections over isolated individual particles. Quantification representative of 2 biological replicates.

In situ hybridisations of *mitfa* in *mitfa*^*vc7*^ background were analysed in Image J software. Channels were split and the green channel analysed. An area of somites/myotome between the lateral and dorsal stripes was selected for analysis, subtract background function was used specifying a rolling ball radius of 10 pixels and disable smoothing. Particle analysis was used to analyze mitfa-positive signal in each embryo (threshold 243/255, 4- infinity pixelsˆ2 size, 0-1 circularity). Mitfa-positive signal as a percentage of area was calculated by: SUM(Ai)/A^t^, where A^i^ is the area of all *mitfa*-positive particles per area selected in an embryo, and A^t^ is the total area selected. Quantification representative of 2 biological replicates.

Quantification of *mitfa*:GFP positive signal was done using ImageJ particle analysis in the green channel (threshold 60/255, 0-infinity, μm^2^ size, 0-1 circularity). Each peripheral nerve was analyzed independently, and GFP-positive signal located at the Dorsal Root Ganglion was excluded. To take into account slight variations in the distance embryos were plated from the bottom of the well, the GFP-positive signal was normalized: the sum of the total are of all GFP-positive particles per peripheral nerve (measured in the green channel) was normalized by the mean peripheral nerve length per fish (measured in the red channel; Tg(*nbt*:dsRED)). Normalized GFP-positive area (pixels^2^) was plotted per peripheral nerve analyzed. Quantification representative of 3 biological replicates.

## Supplemental Information

Document S1. Figures S1–S6

Table S1. DDX21 Mass Spec Peptides, Related to Figure 3

Table S2. DDX21 Phospho-Sites, Related to Figure 3

Table S3. 4sU_4sUpaused_ChIP Data Summary, Related to Figures 4 and 5

Table S4. Enrichment Analysis of DDX21_PRL3 Overlapping Genes, Related to Figure 5

Table S5. gProfiler Drerio All Results, Related to Figure 6

Table S6. GSEA in Patient Cohorts, Related to Figure 7

Table S7. Oligonucleotide Table, Related to STAR Methods

Document S2. Article plus Supplemental Information

## References

[bib1] Ahn J.H., Kim S.J., Park W.S., Cho S.Y., Ha J.D., Kim S.S., Kang S.K., Jeong D.G., Jung S.K., Lee S.-H. (2006). Synthesis and biological evaluation of rhodanine derivatives as PRL-3 inhibitors. Bioorg. Med. Chem. Lett..

[bib2] Al-Aidaroos A.Q., Zeng Q. (2010). PRL-3 phosphatase and cancer metastasis. J. Cell. Biochem..

[bib3] Alonso-Curbelo D., Riveiro-Falkenbach E., Pérez-Guijarro E., Cifdaloz M., Karras P., Osterloh L., Megías D., Cañón E., Calvo T.G., Olmeda D. (2014). RAB7 controls melanoma progression by exploiting a lineage-specific wiring of the endolysosomal pathway. Cancer Cell.

[bib4] Aubin-Houzelstein G., Djian-Zaouche J., Bernex F., Gadin S., Delmas V., Larue L., Panthier J.J. (2008). Melanoblasts' proper location and timed differentiation depend on Notch/RBP-J signaling in postnatal hair follicles. J. Invest. Dermatol..

[bib5] Bai X., Kim J., Yang Z., Jurynec M.J., Akie T.E., Lee J., LeBlanc J., Sessa A., Jiang H., DiBiase A. (2010). TIF1gamma controls erythroid cell fate by regulating transcription elongation. Cell.

[bib6] Bardelli A., Saha S., Sager J.A., Romans K.E., Xin B., Markowitz S.D., Lengauer C., Velculescu V.E., Kinzler K.W., Vogelstein B. (2003). PRL-3 expression in metastatic cancers. Clin. Cancer Res..

[bib7] Basak S., Jacobs S.B., Krieg A.J., Pathak N., Zeng Q., Kaldis P., Giaccia A.J., Attardi L.D. (2008). The metastasis-associated gene Prl-3 is a p53 target involved in cell-cycle regulation. Mol. Cell.

[bib8] Bouché V., Espinosa A.P., Leone L., Sardiello M., Ballabio A., Botas J. (2016). Drosophila Mitf regulates the V-ATPase and the lysosomal-autophagic pathway. Autophagy.

[bib9] Bowman S.L., Bi-Karchin J., Le L., Marks M.S. (2019). The road to lysosome-related organelles: insights from Hermansky-Pudlak syndrome and other rare diseases. Traffic.

[bib10] Budi E.H., Patterson L.B., Parichy D.M. (2011). Post-embryonic nerve-associated precursors to adult pigment cells: genetic requirements and dynamics of morphogenesis and differentiation. PLoS Genet..

[bib11] Cagan R.L., Zon L.I., White R.M. (2019). Modeling cancer with flies and fish. Dev. Cell.

[bib12] Calo E., Flynn R.A., Martin L., Spitale R.C., Chang H.Y., Wysocka J. (2015). RNA helicase DDX21 coordinates transcription and ribosomal RNA processing. Nature.

[bib13] Calo E., Gu B., Bowen M.E., Aryan F., Zalc A., Liang J., Flynn R.A., Swigut T., Chang H.Y., Attardi L.D., Wysocka J. (2018). Tissue-selective effects of nucleolar stress and rDNA damage in developmental disorders. Nature.

[bib14] Camargo-Sosa K., Colanesi S., Müller J., Schulte-Merker S., Stemple D., Patton E.E., Kelsh R.N. (2019). Endothelin receptor Aa regulates proliferation and differentiation of Erb-dependent pigment progenitors in zebrafish. PLoS Genet..

[bib15] Cancer Genome Atlas Network (2015). Genomic classification of Cutaneous Melanoma. Cell.

[bib16] Chazotte B. (2011). Labeling lysosomes in live cells with LysoTracker. Cold Spring Harb. Protoc..

[bib17] Chen F.X., Smith E.R., Shilatifard A. (2018). Born to run: control of transcription elongation by RNA polymerase II. Nat. Rev. Mol. Cell Biol..

[bib18] Chong P.S.Y., Zhou J., Chooi J.Y., Chan Z.L., Toh S.H.M., Tan T.Z., Wee S., Gunaratne J., Zeng Q., Chng W.J. (2019). Non-canonical activation of beta-catenin by PRL-3 phosphatase in acute myeloid leukemia. Oncogene.

[bib19] Cirenajwis H., Ekedahl H., Lauss M., Harbst K., Carneiro A., Enoksson J., Rosengren F., Werner-Hartman L., Törngren T., Kvist A. (2015). Molecular stratification of metastatic melanoma using gene expression profiling: prediction of survival outcome and benefit from molecular targeted therapy. Oncotarget.

[bib79] DeLuca D.S., Levin J.Z., Sivachenko A., Fennell T., Nazaire M.D., Williams C., Reich M., Winckler W., Getz G. (2012). RNA-SeQC: RNA-seq metrics for quality control and process optimization. Bioinformatics.

[bib20] den Hollander P., Rawls K., Tsimelzon A., Shepherd J., Mazumdar A., Hill J., Fuqua S.A., Chang J.C., Osborne C.K., Hilsenbeck S.G. (2016). Phosphatase PTP4A3 promotes triple-negative breast cancer growth and predicts poor patient survival. Cancer Res..

[bib80] Dobin A., Davis C.A., Schlesinger F., Drenkow J., Zaleski C., Jha S., Batut P., Chaisson M. (2013). Gingeras TR. STAR: ultrafast universal RNA-seq aligner. Bioinformatics.

[bib81] Dolinsky T.J., Nielsen J.E., McCammon J.A., Baker N.A. (2014). PDB2PQR: an automated pipeline for the setup of Poisson–Boltzmann electrostatics calculations. Nucleic Acids Res..

[bib21] Dooley C.M., Mongera A., Walderich B., Nüsslein-Volhard C. (2013). On the embryonic origin of adult melanophores: the role of ErbB and Kit signalling in establishing melanophore stem cells in zebrafish. Development.

[bib22] Dooley C.M., Schwarz H., Mueller K.P., Mongera A., Konantz M., Neuhauss S.C., Nüsslein-Volhard C., Geisler R. (2013). Slc45a2 and V-ATPase are regulators of melanosomal pH homeostasis in zebrafish, providing a mechanism for human pigment evolution and disease. Pigment Cell Melanoma Res..

[bib23] Duciel L., Anezo O., Mandal K., Laurent C., Planque N., Coquelle F.M., Gentien D., Manneville J.B., Saule S. (2019). Protein tyrosine phosphatase 4A3 (PTP4A3/PRL-3) promotes the aggressiveness of human uveal melanoma through dephosphorylation of CRMP2. Sci. Rep..

[bib24] Hayward N.K., Wilmott J.S., Waddell N., Johansson P.A., Field M.A., Nones K., Patch A.M., Kakavand H., Alexandrov L.B., Burke H. (2017). Whole-genome landscapes of major melanoma subtypes. Nature.

[bib25] Hultman K.A., Budi E.H., Teasley D.C., Gottlieb A.Y., Parichy D.M., Johnson S.L. (2009). Defects in ErbB-dependent establishment of adult melanocyte stem cells reveal independent origins for embryonic and regeneration melanocytes. PLoS Genet..

[bib26] Hultman K.A., Johnson S.L. (2010). Differential contribution of direct-developing and stem cell-derived melanocytes to the zebrafish larval pigment pattern. Dev. Biol..

[bib27] Iyengar S., Kasheta M., Ceol C.J. (2015). Poised regeneration of zebrafish melanocytes involves direct differentiation and concurrent replenishment of tissue-resident progenitor cells. Dev. Cell.

[bib28] Johnson S.L., Nguyen A.N., Lister J.A. (2011). mitfa is required at multiple stages of melanocyte differentiation but not to establish the melanocyte stem cell. Dev. Biol..

[bib29] Jonkers I., Lis J.T. (2015). Getting up to speed with transcription elongation by RNA polymerase II. Nat. Rev. Mol. Cell Biol..

[bib82] Kharchenko P.V., Silberstein L., Scadden D.T. (2014). Bayesian approach to single-cell differential expression analysis. Nat. Methods.

[bib30] Kobayashi M., Bai Y., Dong Y., Yu H., Chen S., Gao R., Zhang L., Yoder M.C., Kapur R., Zhang Z.-Y., Liu Y. (2014). PRL2/PTP4A2 phosphatase is important for hematopoietic stem cell self-renewal. Stem Cells.

[bib31] Kobayashi M., Nabinger S.C., Bai Y., Yoshimoto M., Gao R., Chen S., Yao C., Dong Y., Zhang L., Rodriguez S. (2017). Protein tyrosine phosphatase PRL2 mediates Notch and kit signals in early T cell progenitors. Stem Cells.

[bib83] Kwan K.M., Fujimoto E., Grabher C., Mangum B.D., Hardy M.E., Campbell D.S., Parant J.M., Yost H.J., Kanki J.P., Chien C.B. (2007). The Tol2kit: a multisite gateway-based construction kit for Tol2 transposon transgenesis constructs. Dev. Dyn..

[bib32] Laurent C., Valet F., Planque N., Silveri L., Maacha S., Anezo O., Hupe P., Plancher C., Reyes C., Albaud B. (2011). High PTP4A3 phosphatase expression correlates with metastatic risk in uveal melanoma patients. Cancer Res..

[bib84] Liao Y., Smyth G.K., Shi W. (2014). featureCounts: an efficient general purpose program for assigning sequence reads to genomic features. Bioinformatics.

[bib33] Lin M.D., Lee H.T., Wang S.C., Li H.R., Hsien H.L., Cheng K.W., Chang Y.D., Huang M.L., Yu J.K., Chen Y.H. (2013). Expression of phosphatase of regenerating liver family genes during embryogenesis: an evolutionary developmental analysis among Drosophila, amphioxus, and zebrafish. BMC Dev. Biol..

[bib34] Liu J., Lichtenberg T., Hoadley K.A., Poisson L.M., Lazar A.J., Cherniack A.D., Kovatich A.J., Benz C.C., Levine D.A., Lee A.V. (2018). An integrated TCGA pan-cancer clinical data resource to drive high-quality survival outcome analytics. Cell.

[bib85] Love M.I., Huber W., Anders S. (2014). Moderated estimation of fold change and dispersion for RNA-seq data with DESeq2. Genome Biol..

[bib35] Maacha S., Planque N., Laurent C., Pegoraro C., Anezo O., Maczkowiak F., Monsoro-Burq A.H., Saule S. (2013). Protein tyrosine phosphatase 4A3 (PTP4A3) is required for Xenopus laevis cranial neural crest migration in vivo. PLoS One.

[bib36] Marie K.L., Sassano A., Yang H.H., Michalowski A.M., Michael H.T., Guo T., Tsai Y.C., Weissman A.M., Lee M.P., Jenkins L.M. (2020). Melanoblast transcriptome analysis reveals pathways promoting melanoma metastasis. Nat. Commun..

[bib37] Maslon M.M., Braunschweig U., Aitken S., Mann A.R., Kilanowski F., Hunter C.J., Blencowe B.J., Kornblihtt A.R., Adams I.R., Cáceres J.F. (2019). A slow transcription rate causes embryonic lethality and perturbs kinetic coupling of neuronal genes. EMBO J..

[bib38] McParland V., Varsano G., Li X., Thornton J., Baby J., Aravind A., Meyer C., Pavic K., Rios P., Köhn M. (2011). The metastasis-promoting phosphatase PRL-3 shows activity toward phosphoinositides. Biochemistry.

[bib39] Mialon A., Thastrup J., Kallunki T., Mannermaa L., Westermarck J., Holmström T.H. (2008). Identification of nucleolar effects in JNK-deficient cells. FEBS Lett..

[bib40] Mohn K.L., Laz T.M., Hsu J.C., Melby A.E., Bravo R., Taub R. (1991). The immediate-early growth response in regenerating liver and insulin-stimulated H-35 cells: comparison with serum-stimulated 3T3 cells and identification of 41 novel immediate-early genes. Mol. Cell. Biol..

[bib41] Möller K., Sigurbjornsdottir S., Arnthorsson A.O., Pogenberg V., Dilshat R., Fock V., Brynjolfsdottir S.H., Bindesboll C., Bessadottir M., Ogmundsdottir H.M. (2019). MITF has a central role in regulating starvation-induced autophagy in melanoma. Sci. Rep..

[bib42] Molleví D.G., Aytes A., Padullés L., Martínez-Iniesta M., Baixeras N., Salazar R., Ramos E., Figueras J., Capella G., Villanueva A. (2008). PRL-3 is essentially overexpressed in primary colorectal tumours and associates with tumour aggressiveness. Br. J. Cancer.

[bib43] Mort R.L., Jackson I.J., Patton E.E. (2015). The melanocyte lineage in development and disease. Development.

[bib44] Newton-Bishop J.A., Beswick S., Randerson-Moor J., Chang Y.M., Affleck P., Elliott F., Chan M., Leake S., Karpavicius B., Haynes S. (2009). Serum 25-hydroxyvitamin D3 levels are associated with breslow thickness at presentation and survival from melanoma. J. Clin. Oncol..

[bib45] Nsengimana J., Laye J., Filia A., Walker C., Jewell R., Van den Oord J.J., Wolter P., Patel P., Sucker A., Schadendorf D. (2015). Independent replication of a melanoma subtype gene signature and evaluation of its prognostic value and biological correlates in a population cohort. Oncotarget.

[bib46] Oskarsson T., Batlle E., Massagué J. (2014). Metastatic stem cells: sources, niches, and vital pathways. Cell Stem Cell.

[bib47] Pearce D.A.N., A J, Freeman T.C., Sims A.H. (2017). Continuous biomarker assessment by exhaustive survival analysis. bioRxiv.

[bib48] Ploper D., Taelman V.F., Robert L., Perez B.S., Titz B., Chen H.W., Graeber T.G., von Euw E., Ribas A., De Robertis E.M. (2015). MITF drives endolysosomal biogenesis and potentiates Wnt signaling in melanoma cells. Proc. Natl. Acad. Sci. USA.

[bib87] Quinlan A.R., Hall I.M. (2010). BEDTools: a flexible suite of utilities for comparing genomic features. Bioinformatics.

[bib49] Rabani M., Levin J.Z., Fan L., Adiconis X., Raychowdhury R., Garber M., Gnirke A., Nusbaum C., Hacohen N., Friedman N. (2011). Metabolic labeling of RNA uncovers principles of RNA production and degradation dynamics in mammalian cells. Nat. Biotechnol..

[bib50] Rambow F., Marine J.C., Goding C.R. (2019). Melanoma plasticity and phenotypic diversity: therapeutic barriers and opportunities. Genes Dev..

[bib51] Rawls J.F., Johnson S.L. (2000). Zebrafish kit mutation reveals primary and secondary regulation of melanocyte development during fin stripe regeneration. Development.

[bib88] Reimand J., Kull M., Peterson H., Hansen J., Vilo J. (2007). g:Profiler–a web-based toolset for functional profiling of gene lists from large-scale experiments. Nucleic Acids Res..

[bib89] Roure A., Rothbächer U., Robin F., Kalmar E., Ferone G., Lamy C., Missero C., Mueller F., Lemaire P. (2007). A multicassette gateway vector set for high throughput and comparative analyses in ciona and vertebrate embryos. PLoS One.

[bib52] Saha S., Bardelli A., Buckhaults P., Velculescu V.E., Rago C., St Croix B., Romans K.E., Choti M.A., Lengauer C., Kinzler K.W., Vogelstein B. (2001). A phosphatase associated with metastasis of colorectal cancer. Science.

[bib53] Sanchez-Vega F., Mina M., Armenia J., Chatila W.K., Luna A., La K.C., Dimitriadoy S., Liu D.L., Kantheti H.S., Saghafinia S. (2018). Oncogenic signaling pathways in The Cancer Genome Atlas. Cell.

[bib90] Sander J.D., Maeder M.L., Reyon D., Voytas D.F., Joung J.K., Dobbs D. (2010). ZiFiT (Zinc Finger Targeter): an updated zinc finger engineering tool. Nucleic Acids Res..

[bib86] Sanner M.F. (1999). Python: a programming language for software integration and development. J. Mol. Graphics Mod..

[bib54] Santoriello C., Sporrij A., Yang S., Flynn R.A., Henriques T., Dorjsuren B., Custo Greig E., McCall W., Stanhope M.E., Fazio M. (2020). RNA helicase DDX21 mediates nucleotide stress responses in neural crest and melanoma cells. Nat. Cell Biol..

[bib55] Sarvi S., Crispin R., Lu Y., Zeng L., Hurley T.D., Houston D.R., von Kriegsheim A., Chen C.H., Mochly-Rosen D., Ranzani M. (2018). ALDH1 bio-activates nifuroxazide to eradicate ALDHhigh melanoma-initiating cells. Cell Chem. Biol..

[bib56] Schnute B., Troost T., Klein T. (2018). Endocytic trafficking of the Notch receptor. Adv. Exp. Med. Biol..

[bib57] Shannon P., Markiel A., Ozier O., Baliga N.S., Wang J.T., Ramage D., Amin N., Schwikowski B., Ideker T. (2003). Cytoscape: a software environment for integrated models of biomolecular interaction networks. Genome Res..

[bib58] Singh A.P., Nüsslein-Volhard C. (2015). Zebrafish stripes as a model for vertebrate colour pattern formation. Curr. Biol..

[bib91] Subramanian A., Tamayo P., Mootha V.K., Mukherjee S., Ebert B.L., Gillette M.A., Paulovich A., Pomeroy S.L., Golub T.R., Lander E.S. (2005). Gene set enrichment analysis: a knowledge-based approach for interpreting genome-wide expression profiles. Proc. Natl. Acad. Sci. U S A.

[bib59] Swift J., Coruzzi G.M. (2017). A matter of time - how transient transcription factor interactions create dynamic gene regulatory networks. Biochim. Biophys. Acta Gene Regul. Mech..

[bib60] Tan J.L., Fogley R.D., Flynn R.A., Ablain J., Yang S., Saint-André V., Fan Z.P., Do B.T., Laga A.C., Fujinaga K. (2016). Stress from nucleotide depletion activates the transcriptional regulator HEXIM1 to suppress melanoma. Mol. Cell.

[bib61] Taylor K.L., Lister J.A., Zeng Z., Ishizaki H., Anderson C., Kelsh R.N., Jackson I.J., Patton E.E. (2011). Differentiated melanocyte cell division occurs in vivo and is promoted by mutations in Mitf. Development.

[bib62] Thura M., Al-Aidaroos A.Q., Gupta A., Chee C.E., Lee S.C., Hui K.M., Li J., Guan Y.K., Yong W.P., So J. (2019). PRL3-zumab as an immunotherapy to inhibit tumors expressing PRL3 oncoprotein. Nat. Commun..

[bib63] Thura M., Al-Aidaroos A.Q.O., Yong W.P., Kono K., Gupta A., Lin Y.B., Mimura K., Thiery J.P., Goh B.C., Tan P. (2016). PRL3-zumab, a first-in-class humanized antibody for cancer therapy. JCI Insight.

[bib64] Tryon R.C., Higdon C.W., Johnson S.L. (2011). Lineage relationship of direct-developing melanocytes and melanocyte stem cells in the zebrafish. PLoS One.

[bib65] van Rooijen E., Fazio M., Zon L.I. (2017). From fish bowl to bedside: the power of zebrafish to unravel melanoma pathogenesis and discover new therapeutics. Pigment Cell Melanoma Res..

[bib66] Wagner D.E., Weinreb C., Collins Z.M., Briggs J.A., Megason S.G., Klein A.M. (2018). Single-cell mapping of gene expression landscapes and lineage in the zebrafish embryo. Science.

[bib67] Wang H., Vardy L.A., Tan C.P., Loo J.M., Guo K., Li J., Lim S.G., Zhou J., Chng W.J., Ng S.B. (2010). PCBP1 suppresses the translation of metastasis-associated PRL-3 phosphatase. Cancer Cell.

[bib92] Wang L., Wang S., Li W. (2012). RSeQC: quality control of RNA-seq experiments. Bioinformatics.

[bib68] Wasmeier C., Hume A.N., Bolasco G., Seabra M.C. (2008). Melanosomes at a glance. J. Cell Sci..

[bib93] Webster D.E., Barajas B., Bussat R.T., Yan K.J., Neela P.H., Flockhart R.J., Kovalski J., Zehnder A., Khavari P.A. (2014). Enhancer-targeted genome editing selectively blocks innate resistance to oncokinase inhibition. Genome Res..

[bib69] Wei M., Korotkov K.V., Blackburn J.S. (2018). Targeting phosphatases of regenerating liver (PRLs) in cancer. Pharmacol. Ther..

[bib70] White R.M., Cech J., Ratanasirintrawoot S., Lin C.Y., Rahl P.B., Burke C.J., Langdon E., Tomlinson M.L., Mosher J., Kaufman C. (2011). DHODH modulates transcriptional elongation in the neural crest and melanoma. Nature.

[bib71] Yang C.T., Johnson S.L. (2006). Small molecule-induced ablation and subsequent regeneration of larval zebrafish melanocytes. Development.

[bib72] Yu G., Wang L.G., Han Y., He Q.Y. (2012). clusterProfiler: an R package for comparing biological themes among gene clusters. Omics.

[bib73] Zeng Q., Dong J.M., Guo K., Li J., Tan H.X., Koh V., Pallen C.J., Manser E., Hong W. (2003). PRL-3 and PRL-1 promote cell migration, invasion, and metastasis. Cancer Res..

[bib74] Zeng Q., Hong W., Tan Y.H. (1998). Mouse PRL-2 and PRL-3, two potentially prenylated protein tyrosine phosphatases homologous to PRL-1. Biochem. Biophys. Res. Commun..

[bib75] Zeng Z., Johnson S.L., Lister J.A., Patton E.E. (2015). Temperature-sensitive splicing of mitfa by an intron mutation in zebrafish. Pigment Cell Melanoma Res..

[bib76] Zhang H., Kozlov G., Li X., Wu H., Gulerez I., Gehring K. (2017). PRL3 phosphatase active site is required for binding the putative magnesium transporter CNNM3. Sci. Rep..

[bib77] Zhang T., Zhou Q., Ogmundsdottir M.H., Möller K., Siddaway R., Larue L., Hsing M., Kong S.W., Goding C.R., Palsson A. (2015). Mitf is a master regulator of the V-ATPase, forming a control module for cellular homeostasis with V-ATPase and TORC1. J. Cell Sci..

[bib78] Zhou L., Ishizaki H., Spitzer M., Taylor K.L., Temperley N.D., Johnson S.L., Brear P., Gautier P., Zeng Z., Mitchell A. (2012). ALDH2 mediates 5-nitrofuran activity in multiple species. Chem. Biol..

