# Supplementary material for: PRL3-DDX21 Transcriptional Control of Endolysosomal Genes Restricts Melanocyte Stem Cell Differentiation
Source: Dev Cell. 2020 Aug 10;54(3):317–332.e9. doi: 10.1016/j.devcel.2020.06.013 (PMC7435699; doi:10.1016/j.devcel.2020.06.013)
Supplement: Document S1. Figures S1–S6 [file mmc1.pdf]

Developmental Cell, Volume *54*

Supplemental Information

PRL3-DDX21 Transcriptional Control of Endolysosomal Genes Restricts Melanocyte Stem Cell Differentiation

Jeanette A. Johansson, Kerrie L. Marie, Yuting Lu, Alessandro Brombin, Cristina Santoriello, Zhiqiang Zeng, Judith Zich, Philippe Gautier, Alex von Kriegsheim, Hannah Brunsdon, Ann P. Wheeler, Marcel Dreger, Douglas R. Houston, Christopher M. Dooley, Andrew H. Sims, Elisabeth M. Busch-Nentwich, Leonard I. Zon, Robert S. Illingworth, and E. Elizabeth Patton

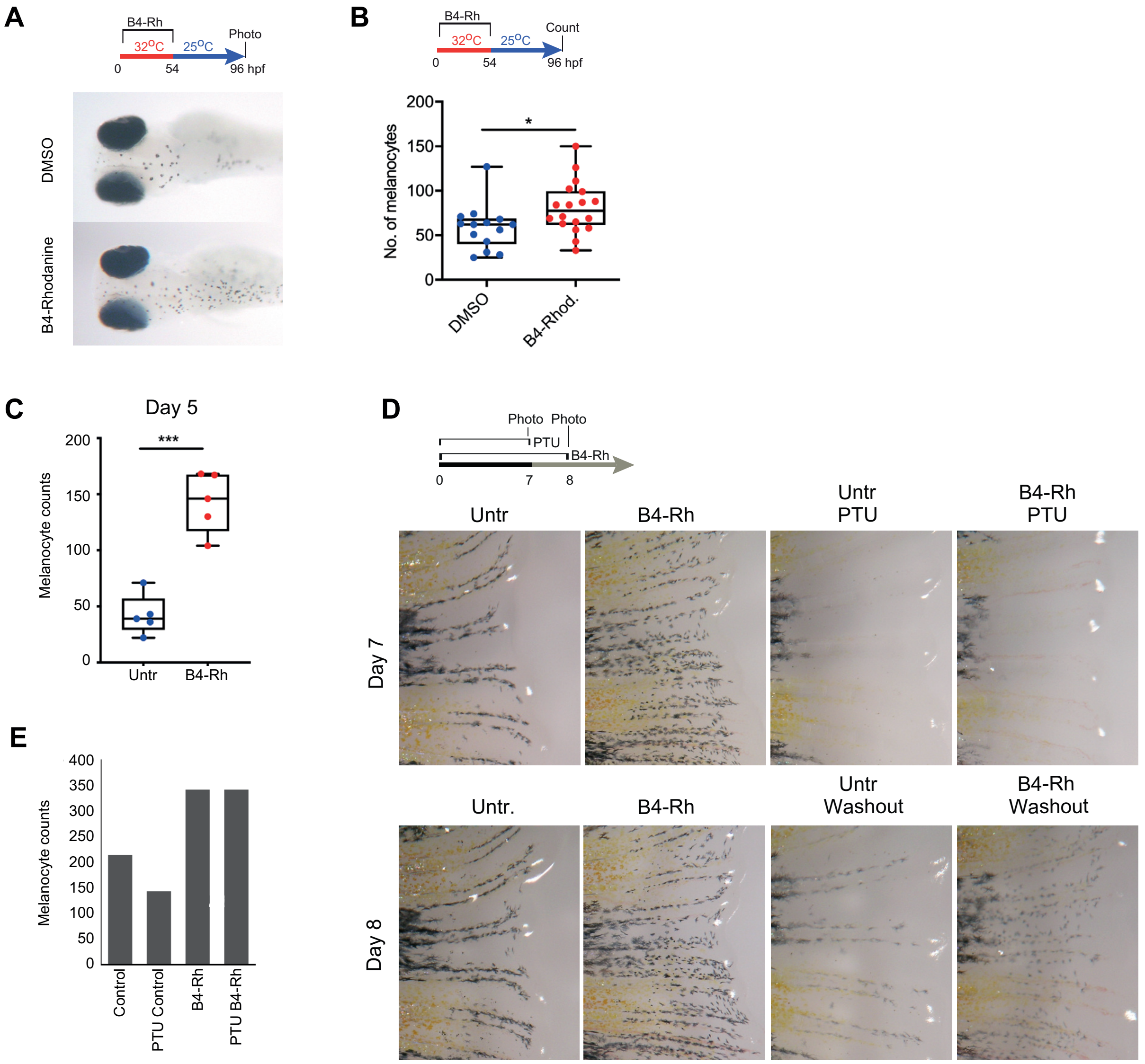

Figure S1, Related to Figure 1.

Figure S1. B4-Rhodanine is an enhancer of MSC regeneration in embryos and adults, Related to Figure 1.

A. Images and **B.** quantification of DMSO and B4-Rhodanine treated *mitfa*^{vc7} embryos in a MSC regeneration assay. Unpaired student's t-test; * p-value=0.0321. n=3 experimental repeats with at least 10 embryos/group

C. Adult tail clips of B4-Rhodanine (1 μM) treated and untreated sibling controls. Melanocyte regeneration at day 5 post tail-clip. Individual melanocytes in the regenerating tail fin tissue were counted. Significance determined by unpaired student's two-tailed t-test (***p-value = 0.0001), n=3 biological replicates.

D, E. Adult tail clips of B4-Rhodanine (1 μM) treated and untreated sibling controls. A tail-clipped fish from each group was co-treated with 1-phenyl-2-thiourea (PTU). Melanocyte regeneration was imaged at days 7 and 8 post tail clip and counted at day 8 post tail clip 24-hours post PTU washout.

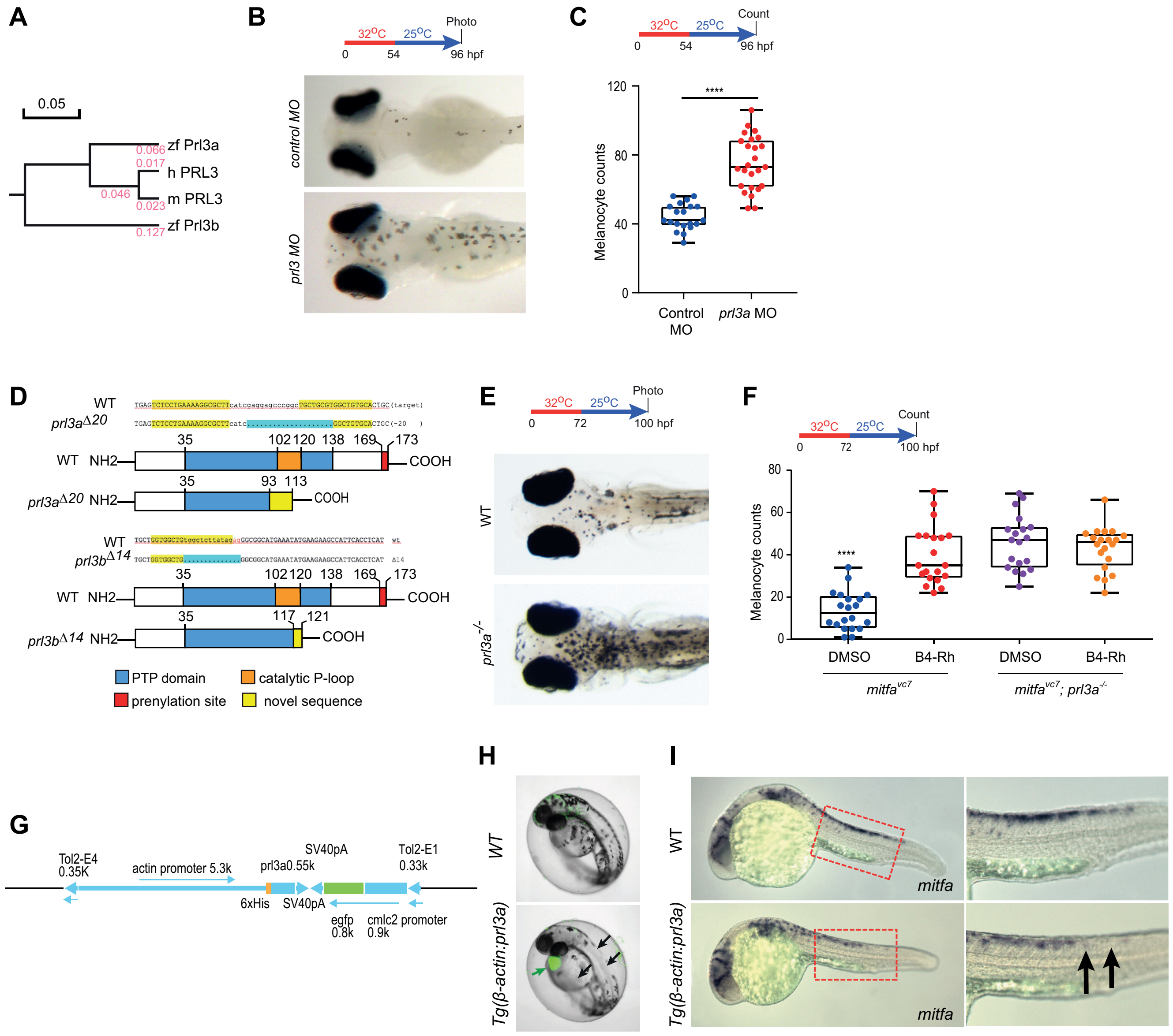

Figure S2, Related to Figure 2.

Figure S2. *prl3a* and *prl3b* zebrafish genetics, Related to Figure 2.

A. Phylogenetic tree of zebrafish (zf), human (h) and mouse (m) PRL3 proteins constructed by DNAMAN.

B, C. Images and quantification of *prl3a* morpholino (MO) knock-down in *mitfa*^{vc7} melanocyte regeneration assay. Student's t-test; **** p-value<0.0001. n=3 experimental repeats, with at least 10 embryos/ group.

D. TALEN and CRISPR-Cas9 genetic mutations and predicted protein structures in *prl3a* and *prl3b* respectively. The TALEN recognition site/ CRISPR guide RNA target sequence is highlighted in yellow. The deleted region is highlighted in blue.

E. Images of *prl3a* mutant and wild type control in *mitfa*^{vc7} temperature regulated melanocyte regeneration.

F. Quantification of melanocytes in the *mitfa*^{vc7} regeneration assay of wild type (WT) or *prl3a* mutant embryos treated with DMSO or 20μM of B4-Rhodanine (B4-Rh) for 72 h prior to initiating regeneration. **** p-value<0.0001; ANOVA using Tukey's multiple comparison test.

G. Schematic image of *prl3a* Tol2 construct introduced into zebrafish.

H. Images of live *Tg(β-actin:prl3a)* transgenic zebrafish embryos and WT sibling control at 48 hpf. Fewer melanocytes and *Tg(cmlc2:eGFP)* heart expression are indicated with black and green arrows, respectively.

I. Images of a whole mount hybridization for *mitfa* of *Tg(β-actin:prl3a)* transgenic zebrafish embryo and wild type sibling control at 24 hpf. N= 12 for each group.

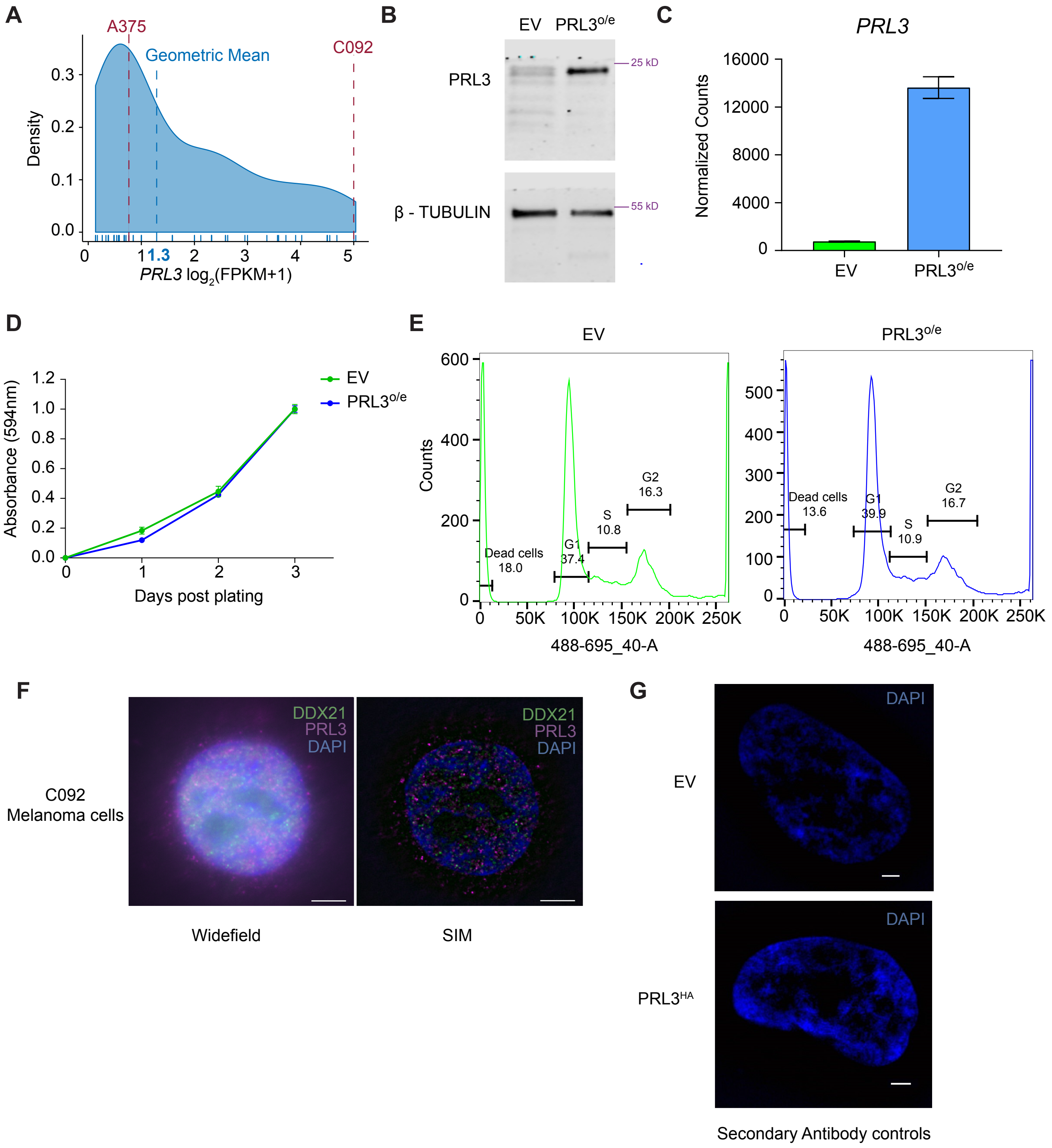

Figure S3, Related to Figure 3.

Figure S3. Characterization of *PRL3* overexpressing cells, and controls for super resolution imaging and data processing, Related to Figure 3.

A. A density plot showing the distribution of *PRL3* expression over different human melanoma cell lines. RNAseq data (FPKM) from a large panel of human melanoma cell lines (45 from Cancer Cell Line Enclopedia and 20 from study EGAS00001000815) were retrieved and plotted. The geometric mean values for *PRL3* across the melanoma cell line panel are annotated on the X-axis.

B. Western blot comparing the levels of PRL3 protein in cells stably expressing the empty vector (EV) or overexpressing PRL3 ($PRL3^{o/e}$) compared with ß-TUBULIN. PRL3 protein is detected with an anti-PRL3 antibody.

C. Normalized counts of *PRL3* transcripts in EV versus PRL3 overexpressing cells using 4sU nascent RNA sequencing data.

D. Growth curve analysis of EV and PRL3 overexpressing cells by SRB assay over a period of 3 days. n=3 biological repeats, 6 technical replicates. Error bars = Standard deviation.

E. FACS cell cycle analysis of EV and PRL3 overexpressing cells. n=2 biological repeats for each cell type. FACS traces are representative for each cell line.

F. Comparison of images without super-resolution and with super-resolution reveal why PRL3 complexes have not been previously detected as foci in the nucleus. Widefield (cells imaged without super-resolution) and SIM images (cells imaged with structured illumination super resolution microscopy) of C092 human melanoma cells that express endogenous PRL3. PRL3 protein in magenta, DDX21 protein in green and DAPI in blue. Scale bars: 5 µm.

G. SIM images of secondary antibody control without primary antibody staining of cells expressing empty vector (EV) and PRL3^{HA} stained cells, DAPI staining in blue. All images were acquired using the same settings. Scale bars: 2μm.

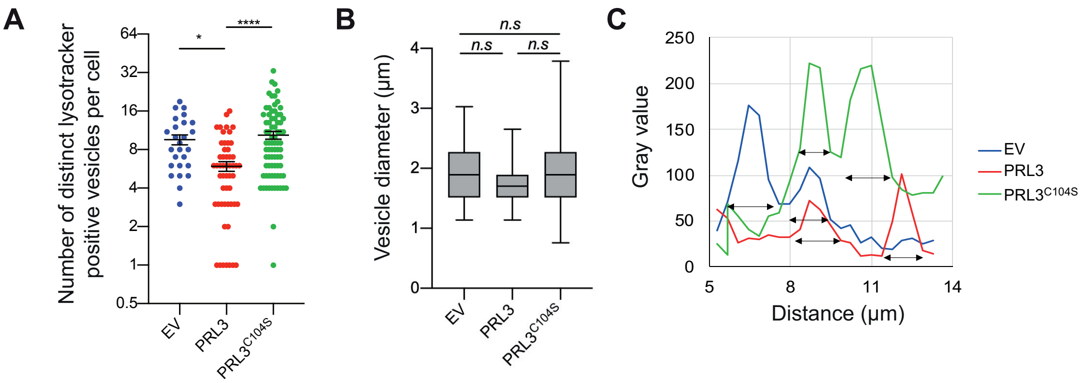

Figure S4, Related to Figure 4.

Figure S4. Lysotracker analysis of vesicles, Related to Figure 4.

A. Quantification of the number of distinct lysotracker-positive particles per cell. Significance determined by ANOVA using Tukey's analysis for multiple comparisons; (* p-value = 0.0119; **** p-value < 0.0001). Line and error bars represent mean and S.E.M.

B. Box plot of vesicle diameters as determined by lysotracker staining and measured by ImageJ. Non-significance between groups was determined by ANOVA using Tukey's analysis for multiple comparisons. Box represents 25th to 75th percentiles, line plotted at median. Whiskers represent Min to Max. Individual vesicles from cells expressing empty vector EV) n= 35; PRL3 n= 40; PRL3(C104S) n=47.

C. Acidic vesicle diameters measured by lysotracker intensity (Gray value) vs. distance (microns). Double headed arrows represent calculated vesicle diameters.

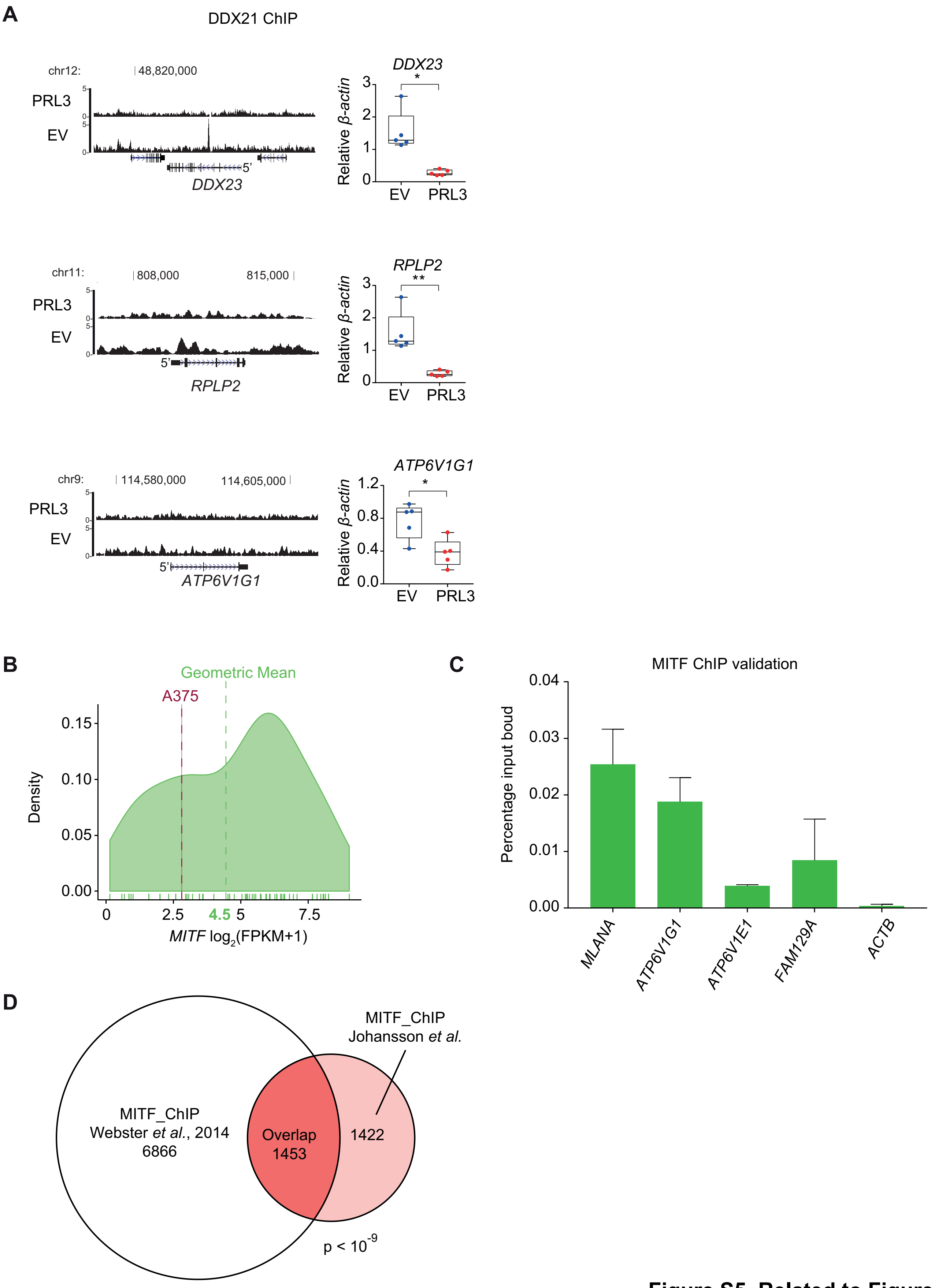

Figure S5, Related to Figure 5.

Figure S5. DDX21 and MITF ChIP-seq controls and analysis, Related to Figure 5.

A. UCSC genome browser tracks of DDX21 ChIP-seq signal at *DDX23*, *RPLP2* and *ATP6V1G1* (left panel) and the corresponding qPCR validation of gene expression between EV and *PRL3*$^{o/e}$ A375 cells (right panel). (*p< 0.05 and **p<0.01; students t-test).

B. Density plot showing the distribution of *MITF* expression over different human melanoma cell lines. RNAseq data (FPKM) from a large panel of human melanoma cell lines (45 from Cancer Cell Line Enclopedia and 20 from study EGAS00001000815) were retrieved and plotted. The geometric mean values for *MITF* across the melanoma cell line panel are annotated as bars on the X-axis. The level of *MITF* expression in the A375 melanoma cell line is indicated.

C. Quantitative PCR analysis of MITF target genes and control gene *ACTB* used for validation of the MITF-ChIP experiment. The graph shows the enrichment ratios between DNA purified from the MITF-ChIP and input DNA. Error bars: SD.

D. Venn diagram shows substantial overlapping gene targets between the two MITF ChIP studies. Webster et al., 2014 MITF ChIP peak information was downloaded from GSE50681 and mapped to Ensembl gene entries by closest distance to perform target gene comparison (p value from Fisher's exact test).

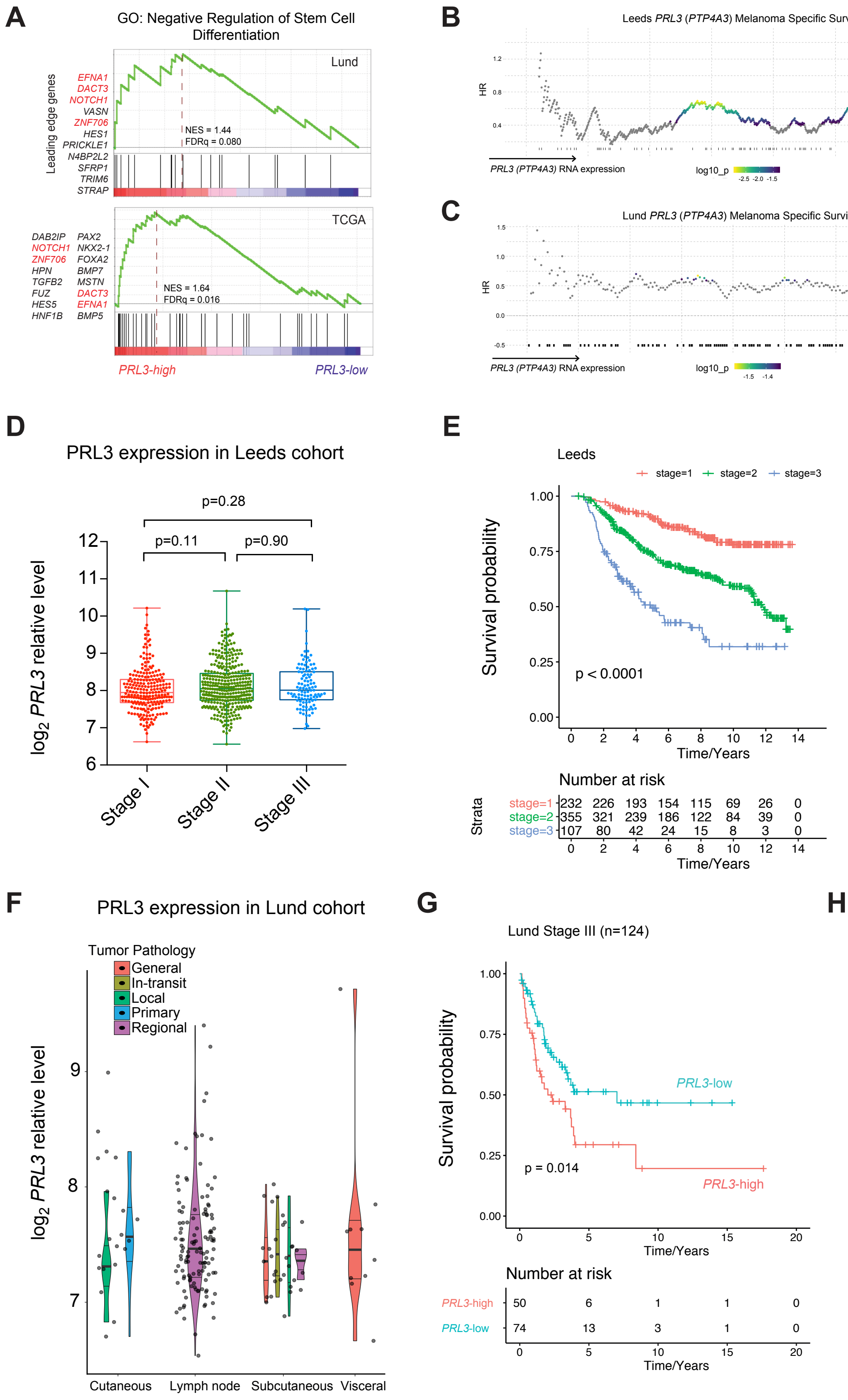

H

GO Terms Enriched in *PRL3-low* Lund Stage III Samples

GO:CC Terms	NES	FDR q-val
LUMENAL_SIDE_OF_MEMBRANE	2.32453	<0.0001
ER_TO_GOLGI_TRANSPORT_VESICLE_MEMBRANE	2.21037	<0.0001
SIDE_OF_MEMBRANE	2.15339	0.00068
PHAGOCYTIC_VESICLE_MEMBRANE	2.11015	0.00089
ENDOCYTIC_VESICLE_MEMBRANE	2.10513	0.00121
PHAGOCYTIC_VESICLE	2.05208	0.00181
ER_TO_GOLGI_TRANSPORT_VESICLE	2.04669	0.00181
PLASMA_MEMBRANE_PROTEIN_COMPLEX	2.03388	0.00192
CLATHRIN_COATED_ENDOCYTIC_VESICLE_MEMBRANE	2.02073	0.00190
CLATHRIN_COATED_VESICLE_MEMBRANE	1.97135	0.00439
TRANSPORT_VESICLE_MEMBRANE	1.92789	0.00705
COATED_VESICLE_MEMBRANE	1.92250	0.00693
CLATHRIN_COATED_ENDOCYTIC_VESICLE	1.90528	0.00776
EXTERNAL_SIDE_OF_PLASMA_MEMBRANE	1.88642	0.00877
ENDOCYTIC_VESICLE	1.87947	0.00879
ENDOSOMAL_PART	1.87930	0.00833
LATE_ENDOSOME	1.86075	0.00948
LYTIC_VACUOLE	1.86068	0.00899
PLASMA_MEMBRANE_RECEPTOR_COMPLEX	1.84888	0.00974
LATE_ENDOSOME_MEMBRANE	1.81242	0.01327
ENDOSOME	1.77423	0.01822
IMMUNOLOGICAL_SYNAPSE	1.76867	0.01860
CLATHRIN_COATED_VESICLE	1.71249	0.03009
VESICLE_MEMBRANE	1.67131	0.04221
COATED_VESICLE	1.67131	0.04058
CYTOPLASMIC_SIDE_OF_MEMBRANE	1.65547	0.04494
CYTOPLASMIC_VESICLE_PART	1.65138	0.04476
LYTIC_VACUOLE_MEMBRANE	1.64323	0.04608
PHOSPHATASE_COMPLEX	1.64258	0.04464

Figure S6, Related to Figure 7.

Figure S6. High expression of *PRL3* is associated with stemness and is an independent predictor of melanoma-specific death at all stages, Related to Figure 7.

A. GSEA plot show PRL3-high melanomas are enriched for genes that inhibit stem cell differentiation. Leading edge genes are indicated with genes shared between Lund and TCGA datasets indicated in red. GO: gene ontology.

B, C. $\log_{10}$ p-value distribution from the optimal cut-off calculation via R package survivALL. Patient samples are ranked by PRL3 (PTP4A3) expression level from low to high. Each patient is represented by a dot in the graph. The cut-point with the lowest p-value is chosen to stratify the patient cohort. All significant cut-points ($p < 0.05$) are highlighted. Each event is shown by the black bar at the bottom x-axis. HR = Hazard Ratio.

D. Beeswarm plot of *PRL3* expression in Leeds cohort samples collected from patients at indicated melanoma stages. P-values calculated using Kolmogorov–Smirnov test.

E. Kaplan-Meier survival curves of melanomas patients diagnosed Stages I, II and III in the Leeds cohort. Later stage diagnosis is associated with worse melanoma-specific survival, $p< 0.0001$, log-rank test.

F. Violin plot of *PRL3* expression in Lund patient samples collected from primary and metastatic tissue sites.

G. Kaplan-Meier survival curves demonstrate that *PRL3-high* melanomas are associated with worse melanoma-specific survival in the Lund Stage III sub-cohort. The PRL3-low/PRL-high groups were determined as in **Figure 7**. $p = 0.014$, log-rank test.

H. Cellular compartment (CC) gene ontology terms enriched in the Lund Stage III PRL3-Low group comparing to the PRL3-High group. All CC enrichment terms relate to vesicle components. NES: normalised enrichment score. FDR q-val: false discovery rate q values.

Supplementary Video legend:

Supplementary Video 1. Regenerating melanocyte develop from deep precursors within the embryo, Related to Figure 1.

Zebrafish embryos treated with NFN1 (left-hand panel) and NFN1 plus B4-Rhodanine (right-hand panel). Regenerating melanocytes can be observed using melanin as a lineage tracer (black). Regenerating melanocytes are observed to develop from deep within the embryos, with the first emerging melanocytes occurring at similar times post-washout, but with many more melanocytes emerging in the B4-Rhodanine treated embryo. Embryo also expresses *Tg(sox10*:*GFP*), and GFP expression was not observed to change between treatment groups.

Supplemental Table Titles

Table S1: DDX21 Mass spec peptides, Related to Figure 3.

Table S2: DDX21 phospho-sites, Related to Figure 3.

Table S3: 4sU_4sUpaused_ChIP data summary, Related to Figures 4 and 5.

Table S4: Enrichment analysis of DDX21_PRL3 overlapping genes, Related to Figure 5.

Table S5: gProfiler_drerio_all_results, Related to Figure 6.

Table S6: GSEA in patient cohorts, Related to Figure 7.

Table S7: Oligonucleotide Table, Related to STAR Methods.
